# An OMICs-based meta-analysis to support infection state stratification

**DOI:** 10.1093/bioinformatics/btab089

**Published:** 2021-02-09

**Authors:** Ashleigh C Myall, Simon Perkins, David Rushton, Jonathan David, Phillippa Spencer, Andrew R Jones, Philipp Antczak

**Affiliations:** Institute of Systems, Molecular and Integrative Biology, University of Liverpool, Liverpool L697ZB, UK; Department of Mathematics, Imperial College London, London SW7 2AZ, UK; Institute of Systems, Molecular and Integrative Biology, University of Liverpool, Liverpool L697ZB, UK; Defence and Security Analysis Division, Defence Science and Technology laboratory (DSTL), Salisbury SP4 0JQ, UK; Chemical, Biological and Radiological Division, Defence Science and Technology laboratory (DSTL), Salisbury SP4 0JQ, UK; Cyber and Information Systems Division, Defence Science and Technology laboratory (DSTL), Salisbury SP4 0JQ, UK; Institute of Systems, Molecular and Integrative Biology, University of Liverpool, Liverpool L697ZB, UK; Institute of Systems, Molecular and Integrative Biology, University of Liverpool, Liverpool L697ZB, UK; Center for Molecular Medicine Cologne, University of Cologne, Cologne 50931, Germany

## Abstract

**Motivation:**

A fundamental problem for disease treatment is that while antibiotics are a powerful counter to bacteria, they are ineffective against viruses. Often, bacterial and viral infections are confused due to their similar symptoms and lack of rapid diagnostics. With many clinicians relying primarily on symptoms for diagnosis, overuse and misuse of modern antibiotics are rife, contributing to the growing pool of antibiotic resistance. To ensure an individual receives optimal treatment given their disease state and to reduce over-prescription of antibiotics, the host response can in theory be measured quickly to distinguish between the two states. To establish a predictive biomarker panel of disease state (viral/bacterial/no-infection), we conducted a meta-analysis of human blood infection studies using machine learning.

**Results:**

We focused on publicly available gene expression data from two widely used platforms, Affymetrix and Illumina microarrays as they represented a significant proportion of the available data. We were able to develop multi-class models with high accuracies with our best model predicting 93% of bacterial and 89% viral samples correctly. To compare the selected features in each of the different technologies, we reverse-engineered the underlying molecular regulatory network and explored the neighbourhood of the selected features. The networks highlighted that although on the gene-level the models differed, they contained genes from the same areas of the network. Specifically, this convergence was to pathways including the Type I interferon Signalling Pathway, Chemotaxis, Apoptotic Processes and Inflammatory/Innate Response.

**Availability:**

Data and code are available on the Gene Expression Omnibus and github.

**Supplementary information:**

Supplementary data are available at *Bioinformatics* online.

## 1 Introduction

The varying differences within both classes of bacterial and viral infections cause the body to respond in a distinct way (Shi and Gewirtz, 2018). Bacteria can be countered by pathways such as complement-mediated lysis and the cell-mediated response for those that survive phagocytosis and live within the cell (intracellular bacteria). In this response, cells present bacterial peptides (antigens) on their surface, which are identifiable by Helper T cells that mediate bacterial destruction (Chaplin, 2010). There are a large variety of viruses and bacteria that affect the host’s immune system in various ways. Whilst some response pathways may overlap for bacterial and viral infections, there are however a number of key differences (Rock *et al.*, 2016; Yewdell and Bennink, 1999). In fact, these different response pathways cause varied transcription (expression) of key genes and, as such, can provide a basis for distinguishing disease state based on the host’s transcriptional response (Manger and Relman, 2000). Such knowledge can be exploited in differentiating between viral, bacterial and control biological states. A previous study demonstrated this by developing a small set of only seven genes that can accurately discriminate bacterial from viral infections across a range of clinical conditions, whilst simultaneously succeeding to determine with high accuracy that patients do not require antibiotics (Sweeney *et al.*, 2016). Simultaneously, there have been numerous other studies looking at diagnosing infection based on the- host’s transcriptional response (Dawany *et al.*, 2014; Hu *et al.*, 2013; Nascimento *et al.*, 2009; Ramilo *et al.*, 2007; Zaas *et al.*, 2009). Previous work failed to generalize as the data contains a far smaller set of pathogens than would be encountered in ‘real world’ scenarios, or studies focussed on single technology platforms, specific pathogens or geographical regions (which contain populations with different HLA alleles and different local pathogen groups). To address this lack of generalization, this work aims to utilize a large-scale analysis over a more representative sample set to improve biomarker generalizability. To gain statistical power and develop more robust panels, meta analyses of publicly available data have proven to be an effective technique (Lagani *et al.*, 2016). However, analysis integrating several cohorts together faces inherent limitations from systematic variations otherwise known as ‘batch effects’. Without proper handling, these batch effects have been demonstrated to be detrimental in population level gene expression analysis (Akey *et al.*, 2007). Data-driven identification of robust biomarkers is a much-debated subject in the biological field. Several machine learning (ML) approaches have been proposed, with typically good performance on datasets used in a given study, but poorer performance when biomarkers are taken forward for validation. This is mainly due to lack of external validation or inherent cross-validation approaches used during the model optimization process. Important is the distinction between uni- and multi-variate approaches to biomarker discovery. While identifying a single predictive marker might be preferred in theory, multi-variate approaches have enabled the discovery of more complex relationships that can provide performance (sensitivity; specificity) far exceeding univariate predictive models (Trevino and Falciani, 2006) including features embedded in specific regions of an underlying molecular interaction network improving biological insight into physiological responses (Ortega *et al.*, 2008). One particular aspect in multi-variate predictive approaches is the optimization of the representative model, which rarely can be achieved through brute force testing and relies on feature selection algorithms. In this publication, we focus on the use of the Random Forest (RF) (Breiman, 2001) classifier, which has been demonstrated to perform well in real-world classification problems with high dimensionality and biased data (Denil *et al.*, 2014). RFs are bagged decision tree models, which classify data points on a subset of features and have been praised for their ability to avoid overfitting (Segal, 2004). Unlike Support Vector Machines or Neural Networks (two frequently used models with high predictive capabilities), RFs forego much of the model selection step using an ensemble approach that builds many weak classifiers into a single strong self-averaging, interpolating model (Cawley and Talbot, 2010). Whilst RFs consist of many weaker models, they have been shown highly effective at capturing non-linear relationships between model predictors and outputs in a number of genomic studies (Díaz-Uriarte and Alvarez de Andrés, 2006; Jiang *et al.*, 2004). Feature selection can vastly improve these ML models by removing and reducing the overall complexity of the data, increasing the statistical power, faster computational implementation and removing the overall noise (Iguyon and Elisseeff, 2003). Various feature selection procedures exist and have been demonstrated in biological problems (Saeys *et al.*, 2007). For this study, we focused on backwards elimination (BW) for gene expression data (Díaz-Uriarte and Alvarez de Andrés, 2006) forming a well-established benchmark, and an evolutionary algorithm, a more explorative and parameterizable search approach, to obtain smaller feature sets (Trevino and Falciani, 2006). BW essentially searches for the optimal feature set by progressively eliminating the least important features from a given dataset and testing whether the new model is significantly more accurate than the previous. Whereas evolutionary algorithms are based on evolving population(s) of models, which are repetitively intermixed, and subject to random point mutations. This evolutionary process is assumed to produce converging model populations in terms of performance and their associated feature sets (de la Fraga and Coello Coello, 2011). In this publication, we focus on the development of predictive models able to distinguish viral, bacterial and no infection samples using publicly available transcriptomics data (human blood samples where individuals had bacterial, viral or no infection) from two microarray technologies (Affymetrix and Illumina). We applied a BW and evolutionary algorithm to these data to identify models predictive of infection status and compared the results in a biological context by exploring the neighbourhoods of these genes. These network representations show that while the technologies develop different models, selection occurs in similar functional space, highlighting the robustness of our models. We further validated our models by evaluating the top models across the two technologies.

## 2 Materials and methods

### 2.1 Data integration

To identify and validate a panel of biomarkers able to differentiate bacterial and viral infections, we performed a meta-analysis of GEO gene expression data, all from open source microarray human blood infection studies. Our analysis was divided into three major method steps: (i) pre-processing, (ii) feature selection and (iii) inferring a gene interaction network to discover and validate gene lists (1). Following the major steps, we performed and report the results of a final out-of-sample test on data not previously used in the training phase for greater validation. All code is available on github (https://github.com/PGB-LIV/Classifying-disease-state-in-high-dimensional-data).

#### Data

Datasets from Affymetrix and Illumina platforms, consisting of 3868 samples, from 21 different studies were included in the analysis (Table 1—available on GEO under GSE162329 and GSE162330). Selection criteria included study set size, class pathogen strain distribution and ability to integrate the data. Studies for which there were ambiguous annotations (possible bacterial (b?) viral (v?)) were incorporated (an analysis for confirmed cases only is shown in Section S2, Supplementary Material). To integrate the data, ProbeIDs were substituted by their gene mappings and deduplicated by selecting the ProbeID/gene combination with the highest average intensity across samples (Table 2 and Wang *et al.*, 2012). Data from each manufacturer was batch-corrected to remove inter-platform and intra-platform batch effects using ComBat (Johnson *et al.*, 2007) in a two-step sequential batch correction pipeline (Section S1, Supplementary Material). For intra platform batch correction, ‘study ID’ was passed as the batch and ‘sample classes’ were used as covariates. For the inter platform batch correction, ‘series’ (platform GPL) was provided as the batch variable with no additional covariates. Batch correction success was estimated by calculating the significance of the overlap of differential gene expression results pre and post batch as well as through principal component analysis (PCA) (Pearson, 1901).

**Table 1.** Summary of platform-level Affymetrix and Illumina datasets prior to pre-processing

Manufacturer	Affymetrix	Affymetrix	Affymetrix	Illumina
Platform (GPL)	GPL570	GPL571	GPL9188	GPL10558
Studies (GSE)	GSE49954, GSE50628, GSE54992, GSE25504, GSE66099, GSE69606, GSE6269, GSE18090, GSE28750, GSE34205	GSE52428, GSE95104, GSE17156	GSE30550	GSE29385, GSE32707, GSE37250, GSE40396, GSE60244, GSE64456, GSE68310
Distinct Genes	22,213	13,383	13,383	19,947
Sample Count (%)	615 (100)	834 (100)	268 (100)	2151 (100)
Bacterial (b) (%)	27 (4.4)	60 (7.2)	0 (0)	215 (10.0)
Uncertain Bacterial (b?) (%)	227 (36.9)	0 (0)	0 (0)	141 (6.6)
Viral (v) (%)	164 (26.7)	358 (42.9)	132 (49.3)	1069 (49.7)
Uncertain Viral (v?) (%)	0 (0)	348 (41.7)	119 (44.4)	0 (0)
Control (c) (%)	156 (25.4)	68 (8.2)	17 (6.3)	467 (21.7)
Other (%)	41 (6.7)	0 (0)	0 (0)	259 (12)

**Table 2.** Merged and batch corrected modelling dataset description

Dataset	Distinct genes	Platforms	Bacterial samples	Viral samples	Control samples	Total samples
Affy_I	13,383	GPL570, GPL571, GPL9188	314 (18.74%)	1121 (66.89%)	241 (14.38%)	1676
Illumina_I	19,947	GPL10558	356 (18.82%)	1069 (56.50%)	467 (24.68%)	1892

Merged and batch corrected Affymetrix and Illumina (ambiguous classes integrated) dataset breakdown by distinct genes, platforms, class make up, and sample count.

#### Feature selection

Two feature selection procedures: (i) a Backward Elimination process (Huang *et al.*, 2009) and (ii) a genetically inspired search algorithm (GALGO) (Trevino and Falciani, 2006) were used. Both search procedures operated using the RF Classifier, implemented in the R Ranger package (Wright and Ziegler, 2017). Datasets were fed into these approaches with their full class list (bacterial/viral/no infection) and a single predictive model requested. Depending on the feature selection strategy, this included different steps described below. For both a study aware data split and smaller class penalty, as implemented in Breiman’s (2001), was used to ensure best possible model development. In both cases, the reported results are based on the evaluation data split.

#### Backward elimination

A 60/20/20 training/test/evaluation data split was used in BW, with 60 used for model training, 20 used to select trained models, then a final 20 as a ‘held out’ subset for final evaluation and reporting, a standard technique in ML (Hastie *et al.*, 2009). For each dataset, we ran 240 BW search procedures, using out-of-bag (OOB) error as the minimization criterion and implementation using the VarSelRF R package (Díaz-Uriarte, 2007). Each run generated a single optimal model that minimized OOB. For each dataset, a single representative model was selected from the 240 runs that maximized accuracy on test data.

#### Genetic algorithm

The Genetic algorithm (GA) optimized approach is an efficient method for creating suitable multivariate models. We used the R library GALGO (Trevino and Falciani, 2006) to identify a small feature model by continuously crossing a number of small feature models (chromosomes of features) with each other, hypothetically identifying better models with successive generations and repeating this procedure several times. We used an initialized fitness goal of 0.95, model size (chromosome size) of 15 genes and *k*-fold cross-validation to counter overtraining. Similar to the BW approach, GALGO uses a multiple split strategy (Trevino and Falciani, 2006). Two hundred and fifty models were generated for each dataset and a representative model established through a frequency based forward selection strategy that ensures only genes that contributed to predictions are included in the final model (Section S2, Supplementary Material).

### 2.2 Inferring underlying interaction network

**Fig. 1.**
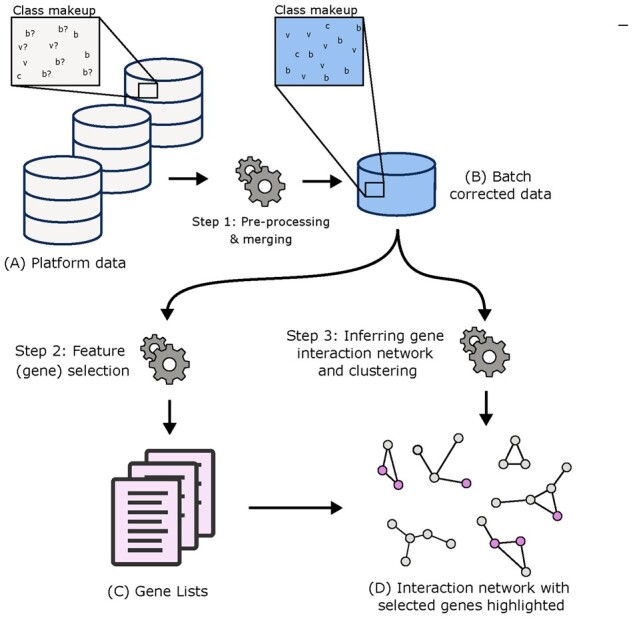
Conceptual overview. Individual data (A), containing bacterial (b), viral (v), control (c), and samples with lower levels of study confidence(? s) are merged. (B) Combined and batch corrected dataset. Feature selection is performed on data B in Step 2 using (i) BW and (ii) an Evolutionary algorithm. (C) Gene Lists obtained in the feature selection. Data B is also used to infer and cluster a gene interaction network by (i) reverse engineering the gene interaction network and (ii) clustering the adjacency matrix. (D) The clustered interaction network overlaid with genes found in the best performing model of each dataset and search procedure

Gene regulatory networks were developed using ARACNe (Margolin *et al.*, 2006) (Fig. 1). To select significant interactions within our dataset, we used a *P*-value threshold < 0.05 in the ARACNe procedure. Networks were loaded into Cytoscape (Shannon *et al.*, 2003) and visualized. To identify highly interconnected sub-networks within our reconstructed regulatory network, we utilized the Cytoscape clustering plugin GLay (Su *et al.*, 2010) to implement the divisive Girvan–Newman algorithm that removes edges based on betweenness (Newman, 2006). This resulted in a number of smaller sub-networks and allowed us to inspect their functional roles within the larger network. DAVID was used to map higher-level ontologies on these subnetworks (Huang *et al.*, 2007). For clusters of genes with enriched and significant terms related to the immune response, we labelled them manually as functionally relevant (FR) clusters. These FR clusters allowed us to make inferences about which biological functions hold predictive power by overlaying model selected genes onto our labelled gene regulatory network.

### 2.3 Out of sample testing

To validate the models obtained by feature selection within the Affymetrix and Illumina datasets, we tested their predictive ability in the other dataset. Briefly, in the case of the Affymetrix optimized model, we extract the best performing genes and retrained and tested the RF classifier using the Illumina dataset with a 60/40 training/test split. For an Illumina optimized model, we followed the same principle but on the Affymetrix dataset. These non-discovery datasets contained samples from different studies and technology and therefore represented the ideal validation datasets. With similar error between discovery and non-discovery data, one can be confident that models have not overfitted to a given dataset and are suggested to be generalizable.

## 3 Results

### 3.1 Integrating data across multiple platforms

The final datasets contained 19,947 and 13,383 distinct genes for the Illumina and Affymetrix datasets, respectively. The lower Affymetrix count was due to platforms GPL571 and GPL9188 that only contained 13,383 genes (Table 1). Manufacturer relevant datasets were merged successfully (Fig. S2, Supplementary Material). The resulting two datasets Affy_I and Illumina_I contained 1676 and 1892 samples, respectively. Both datasets contained more than 50% viral samples with bacterial samples the most underrepresented class (Table 1).

### 3.2 Identifying biomarker panels predictive of viral, bacterial and no-infection

A backward selection (BW) and genetic algorithm-based approach (GA) were applied to the resulting data. To compare the selection strategies between the two approaches, genes were ranked and their relative gene selection frequencies computed (Table 3). BW search procedures in both technologies converged to a small set of genes. For Affymetrix, 14 genes were included at a rate of 1.0, whereas, for Illumina BW, results contain 12 genes at a rate of 1.0 (Table 3). GA’s on the other hand contained a much wider gene selection in the evolved chromosome; in both manufacturers, only a single gene was included at a relative rate of 1.0. Overall search results (aggregated between runs by frequency) from BW and GA in both Affymetrix and Illumina all contained LY6E (lymphocyte antigen 6E, UniProt: Q16553) amongst their nine most frequently selected genes. IFI27 (interferon alpha-inducible protein 27, mitochondrial, UniProt: P40305) and IFI44 (interferon-induced protein 44, UniProt: Q8TCB0), also had high selection frequencies for three of the four search procedures (Table 3). These three genes (LY6E, IFI27 and IFI44) are all type-I interferon-inducible genes (ISGs), demonstrated to have altered expressions in disease states and known to be highly effective at countering infection (Kyogoku *et al.*, 2013; McNab *et al.*, 2015; Rönnblom and Eloranta, 2013; Schneider *et al.*, 2014). Many of the other frequently selected genes have been previously linked to disease state in literature. MS4A4A, IFI44L, OAS2 and IFIT5 are known ISGs; increased levels of MMP8 have been observed in HIV viral studies (Singh *et al.*, 2018); SIGLEC1, a Type 1 transmembrane protein, is expressed by a subpopulation of macrophages found upregulated during in vivo respiratory syncytial virus infections (Jans *et al.*, 2018) and contributes to the initiation of formation of the virus-containing compartment (Hammonds *et al.*, 2017).

**Table 3.** Top 16 Gene selection for Affymetrix and Illumina models and their relative selection frequencies

Affymetrix genes (relative frequency)		Illumina (relative frequency)	
BW	GA	BW	GA
MS4A4A (1.00)	PCOLCE2 (1.00)	IFI44 (1.00)	IFI27 (1.00)
MTHFD2 (1.00)	CEP55 (0.97)	MCEMP1 (1.00)	EPSTI1 (0.41)
RSL24D1 (1.00)	HBA1.HBA2 (0.88)	CD177 (1.00)	LY6E (0.39)
TSPO (1.00)	CDC27 (0.66)	GPR84 (1.00)	SPATS2L (0.34)
LY6E (1.00)	TSPO (0.56)	EIF1 (1.00)	RSAD2 (0.26)
MMP8 (1.00)	LY6E (0.50)	IFI27 (1.00)	IFIT5 (0.24)
NSUN7 (1.00)	MMP8 (0.47)	EPSTI1 (1.00)	IFI44 (0.24)
IFI27 (1.00)	PGD (0.47)	REPIN1 (1.00)	ZDHHC19 (0.22)
CXCL10 (1.00)	RSL24D1 (0.47)	LY6E (1.00)	FCGR1A; FCGR1CP (0.21)
ITGAM (1.00)	SIGLEC1 (0.47)	ALKBH5 (1.00)	IFI44L (0.19)
PSMA6; KIAA0391 (1.00)	IFI44 (0.44)	EEF2 (1.00)	MCEMP1 (0.19)
GRB10 (1.00)	OAS3 (0.44)	RBM33 (1.00)	PRC1 (0.18)
GYG1 (1.00)	WNT10B (0.44)	ARRB1 (0.99)	HPGD (0.17)
PGD (1.00)	ADAMTS3 (0.41)	DSCR3 (0.99)	OAS2 (0.17)
CD177 (0.99)	HPR.HP (0.38)	TSPAN18 (0.99)	HERC5 (0.17)
OLAH (0.99)	OLAH (0.38)	FCGR1A; FCGR1CP (0.96)	IFITM3 (0.15)

Frequency provided in brackets is based on the model selection frequency in each optimization run (the number of times a gene was selected across the number of optimised models). Bold genes are included amongst three of models top 16 selection, and underlined genes are included in all four.

**Fig. 2.**
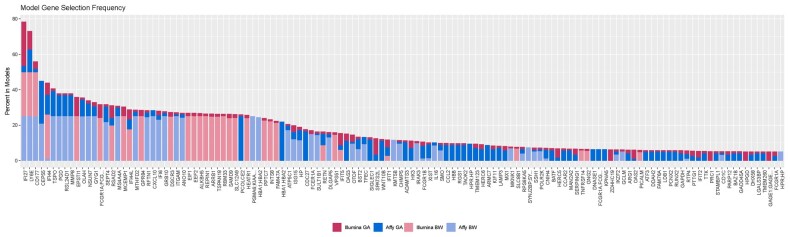
Gene frequency in Affymetrix and Illumina models. Each model frequency is scaled between 1 and 25. Model overlapping gene frequencies are then stacked and coloured by model-dataset combination. Affymetrix models by shades of blue and Illumina models by shades of red

**Fig. 3.**
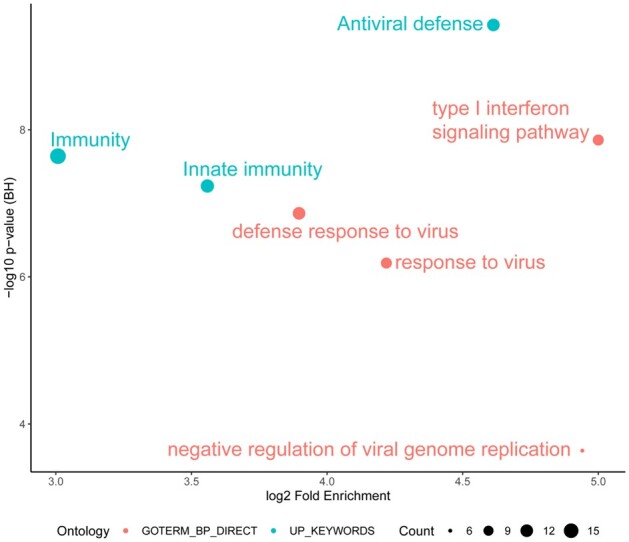
Functional enrichment analysis of the identified 88 genes intersecting between Affymetrix and Illumina search procedures. ‘Antiviral defense’ is the most significant term, whilst ‘type I interferon signalling pathway’ is the most enriched albeit with a non-significant *P*-value

To further investigate gene convergence, we compared the relative model gene inclusion rates for all search procedures together. Figure 2 shows the resulting stacked frequency, where 88 genes are visualized that had greater than 5% aggregated inclusion across all search procedures (Table S38, Supplementary Material). This highlighted LY6E, IFI27 and CD177 as important key genes. CD177 is a neutrophil-specific receptor known to be at increased expression for patients in septic shock (Demaret *et al.*, 2016; Stroncek, 2007). To better compare the models, we performed a functional enrichment analysis of these 88 intersecting genes between the two manufacturers’ models. We found both highly enriched and significant terms relating to the immune response: ‘Antiviral defense’ comprising 12 genes, the ‘type I interferon signalling pathway’ that included 10 genes, and ‘Immunity’ encompassing 17 genes (Fig. 3). Final representative models were developed (Affy_BW, Affy_GA, Illumina_BW and Illumina_GA) and evaluated on their performance on a held-out data split. Model performance was recorded as the size of the gene list and its class-based performance in terms of: Balanced Accuracy, Sensitivity, Specificity and Mcnemar’s Test *P*-value that tests for consistency in responses and can reveal bias to classifying a certain class (Dietterich, 1998) (full results included in S2 Biomarker search results, Supplementary Material). Average model size was similar between both Affymetrix and Illumina models (30–37 genes) (Table 4). On average models classified 0.89 of Bacterial, 0.72 of Control and 0.86 of Viral classes correctly across all datasets. In particular, the Affymetrix models, BW and GA, performed particularly well in terms of balanced accuracy on bacterial samples (0.94 and 0.93, respectively). In terms of sensitivity all models performed well for bacterial and viral classes (on average 0.85 and 0.93, respectively), however control sample performance was worse when compared to the viral and bacterial classes (0.57). Evaluating model specificity, bacterial classification performance was particularly high over all models (averaging 0.95) that would suggest that we can identify bacterial samples particularly well regardless of the model used.

**Table 4.** Overall optimal model performance

	Affymetrix		Illumina		Average
	BW	GA	BW	GA	
Gene-set size	33	36	30	37	34
Balanced accuracy (B/C/V)	0.94/0.78/0.86	0.93/0.82/0.89	0.86/0.70/0.78	0.82/0.58/0.89	0.89/0.72/0.86
Sensitivity (B/C/V)	0.90/0.57/0.97	0.88/0.66/0.97	0.80/0.47/0.87	0.83/0.58/0.89	0.85/0.57/0.93
Specificity (B/C/V)	0.93/0.96/0.76	0.99/0.97/0.81	0.93/0.92/0.87	0.93/0.94/0.77	0.95/0.95/0.80
Mcnemar’s test *P*-value	3.57E-03	4.90E-10	2.36E-03	4.33E-15	5.93E-03

### 3.3 Inferred interaction networks

**Fig. 4.**
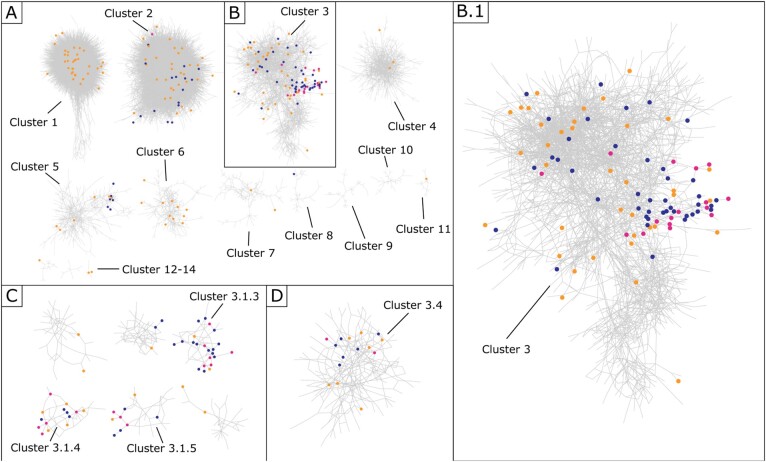
Clustered Illumina interaction network. Illumina models’ selected genes are blue, Affymetrix selected genes are orange, and those intersecting both technologies are pink. (A) Illumina Interaction network after initial clustering (visualizing clusters > 10 genes). (B) Cluster 3, containing the most selected genes that intersected between Affymetrix and Illumina models. (B.1) Cluster 3 enlarged. (C) Highly selected sub clusters of Cluster 3. (D) Cluster 3.4, a sub cluster of Cluster 3 containing two genes that were selected by both Affymetrix and Illumina models

GLay clustering of the Illumina gene interaction network initially revealed 14 clusters containing more than 10 genes (Fig. 4—see Section S3, Supplementary Material, for the Affymetrix based analysis). To enable a more granular analysis of specific network sections (those indicated to be FR in the immune response as indicated by enrichment analysis, or containing genes selected by our models) we further partitioned several of the initial clusters, forming a network hierarchy (limited to a depth of 3). This resulted in 110 distinct groups of genes that we analysed (Table 5). In the Illumina data-derived results, 24 of the 110 clusters were marked as FR (Table 5), of these, 10 FR clusters contained genes selected by an Illumina optimal model. In total 55 genes from the Illumina optimal models were found in these 10 FR clusters (68% of all the 81 Illumina model selected genes found in the network). Additionally, a small number of clusters (four) were selected by every optimal Illumina model.

**Table 5.** Illumina interpreted inferred interaction network properties

Nodes (genes)	Sub-clusters of more than four genes (% of all)	FR clusters (% of all)	FR clusters selected by > 1 model (% of all)	FR clusters selected by all four models (% of all)
19839	110 (1.00)	24 (21)	10 (9)	4 (4)

Clusters have been labelled either functionally related to the immune response (FR). For a cluster to be labelled as FR, functional enrichment analysis of their gene list will have revealed terms both enriched and significant implicated in the host response to disease.

#### Affymetrix—illumina cluster comparison

We found a similar number of clusters converged between both Affymetrix and Illumina-derived gene lists in their respective networks (Section S3, Supplementary Material). Importantly, the clusters were identified using an approach that tests for increased connectivity within the network, and as such, modules containing highly co-expressed genes are identified. Separate clusters therefore represent features that are further away from each other. The observed convergence therefore suggests that the RF models are selecting features from particular gene functional units within our network. Interestingly, the gene level convergence is more heterogenous likely due to technical differences between the technologies. For greater biological understanding we compared the most selected clusters from both the Affymetrix and Illumina Interaction Network. In Illumina this was Cluster 3.1.3 (Section S3, Supplementary Material). Whilst the size between both clusters was not comparable (Affymetrix—Cluster 5 being 435 Genes and Illumina Cluster 3.1.3 being only 47) we found an intersection of 16 Genes (DDX60, IFI35, IFI44, IFI44L, IFIH1, IFIT1, IFIT2, IRF7, ISG15, MX1, OAS2, SCO2, TIMM10, TRAFD1, TRIM22 and ZBP1), which was statistically significant (*P*-value < 3.18e-12), 10 of which known to be ISGs (IFI35, IFI44, IFI44L, IFIH1, IFIT1, IFIT2, IRF7, ISG15, MX1, OAS2) (McNab *et al.*, 2015). Performing DAVID enrichment analysis on both clusters, we find in Illumina Cluster 3.1.3 one highly enriched term ‘type I interferon signalling pathway’ albeit with a non-significant *P*-value (Section S3, Supplementary Material). We do not see the same term in the Affymetrix cluster; however, it does contain numerous ISGs, which we saw commonly amongst gene lists. This convergence between independent feature selection across separate manufacturers and different studies reinforces the high predictive power of ISGs for discriminating disease state across infection studies.

#### Independent cluster convergence between affymetrix and illumina models

To examine whether convergence between Affymetrix and Illumina was also to the same clusters containing the same genes we looked at where in the Illumina interaction network Affymetrix gene lists selected from (Fig. 4, full break down in Section S3, Supplementary Material). Although selected genes varied between Affymetrix and Illumina sets, we indeed found that both converged around the same clusters of genes. Moreover, we found that 19 clusters (including lower level sub clusters) were selected by both Affymetrix and Illumina models in the Illumina interaction network. Interestingly amongst this set, the four sub clusters intersecting across all Illumina gene lists (all from within the larger Illumina-Cluster 3: Fig. 4) were also selected by Affymetrix gene lists: Illumina-Cluster 3.1.3, Illumina-Cluster 3.1.4, Illumina-Cluster 3.1.5 and Illumina-Cluster 3.4. All of these clusters contained genes revealed by selection frequency analysis in previous Section 4.2. We investigated all four clusters selected by all Illumina models (Clusters 3.1.3, 3.1.4, 3.1.5 and 3.4) and found they could be separated functionally to different aspects of an immune response. As mentioned, enrichment analysis on Illumina Cluster 3.1.3 revealed the ISGs to be present. However, enrichment analysis also revealed a number of both highly enriched and significant terms related to viral infections (‘response to Viruses’, ‘defense response to virus’), and most prominently ‘Antiviral Defense’, which is no surprise given the high number of interferon related genes in the cluster (Section S3, Supplementary Material). Comparing the 47 genes in Clusters 3.1.3 to our model frequency analysis revealed 18 overlapping genes (DHX58, EPSTI1, HERC5, IFI44, IFI44L, IFI6, IFIT1, IFIT2, IFIT5, ISG15, MX1, OAS2, OAS3, RSAD2, RTP4, SAMD9, SPATS2L and TMEM123). For cluster 3.1.4, in which LY6E resides, it bears relation to cell signalling with by far the most significant and enriched term ‘chemotaxis’ (Section S3, Supplementary Material). Chemotaxis is well known to play critical role in host response to infections and is specifically involved in recruitment of leukocytes and movement of lymphocytes around the body (Jin *et al.*, 2008). The intersect of cluster 3.1.4 with our model frequency analysis was also large, being 12 of its 40 genes (ATF3, CCL2, CXCL10, HERC6, LAMP3, LGALS3BP, LY6E, OTOF, PARP12, SEPT4, SERPING1 and SIGLEC1). Cluster 3.1.5 contains genes involved in programmed cell death, containing several significant and enriched terms like ‘Apoptosis’, ‘Regulation of apoptotic process’ and ‘apoptotic process’ (Section S3, Supplementary Material). A total of 3 of its 37 genes intersected our model frequency analysis (CHMP5, FCGR1A and FCGR1B). Illumina cluster 3.4 contained genes more related to general innate responses with enriched terms containing ‘Inflammatory response’ and ‘innate immune response’ with non-significant *P*-values (Section S3, Supplementary Material). Amongst the genes are a number related to the Toll-like receptor family (also an enriched and significant term), which respond to microbial products and viruses, and are key receptors of the innate immune system (Das *et al.*, 2017). Although not visible in the functional enrichment analysis, Illumina Cluster 3.4 also contained a number of Interleukin genes (IL1B, IL1R1, IL4R, IL18R1, IRAK3), known to be involved in inflammation and fundamental to innate immunity (Dinarello, 2011). Out of the 253 genes in cluster 3.4, 15, including CD177, intersected with previous frequency analysis (BATF, CD177, DDAH2, GADD45A, GPR84, GRB10, GYG1, HK3, IRAK3, MAN2A2, MKNK1, NSUN7, SULT1B1, TSPO and ZDHHC19).

### 3.4 Cross manufacturer gene list performance

**Fig. 5.**
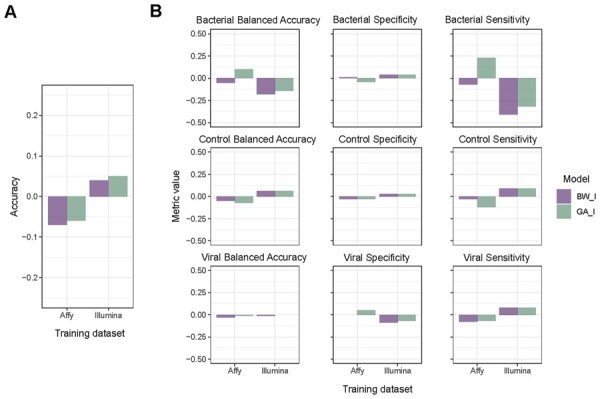
Cross manufacturer model change in performance. Difference in performance when taking Affymetrix-derived models and testing on the Illumina data, and the Illumina-derived models when testing on the Affymetrix data. (A) Difference in performance in terms of overall accuracy. (B) Class-based performance in terms of balanced accuracy, sensitivity, and specificity. For each performance measure, bars are grouped by model, and each bar refers to the difference between performance on the original dataset (which each model was discovered on) and the performance on the data it had not been exposed too. For Affymetrix models, this would contrast the performance on the Affymetrix data, with the same model’s performance on the Illumina data

We evaluated each of the BW & GA representative models from Affymetrix on the Illumina Data and Illumina Models on the Affymetrix data. Contrasting each model’s performance between these two discovery and non-discovery datasets we get the performance results depicted in Figure 5. This figure shows the difference between overall accuracy and class-based accuracy, speciality and sensitivity when generalizing our models to data pertaining from a different technology and set of studies. In terms of overall accuracy (Fig. 5A) Affymetrix models, both GA ad BW, performed worse when applying to the Illumina data. However, the drop was less than 0.1 for both Affymetrix GA and BW. Whereas for Illumina, both GA and BW models slightly gained accuracy when applied to the Affymetrix data (0.04 and 0.05, respectively). Looking specifically at bacterial performance (Fig. 5B), both Illumina models performed worse on the Affymetrix data in terms of bacterial balanced Accuracy (BW_I 0.71 and GA_I 0.73 2dp). Whereas the Affymetrix models performed well on the Illumina data (BW_I 0.89 and GA_I 0.89 2dp). In terms of bacterial specificity there was little change for all models, staying within ± 0.05 2dp of change in performance. However, in terms of bacterial sensitivity, the Illumina models performed particularly worse on the Affymetrix data (BW_I 0.44 and GA_I 0.47 2dp). Across viral class specific metrics (Fig. 5B), no model had any large change in Balanced Accuracy (change < 0.05 2dp). The largest metric change was seen in sensitivity, with Affymetrix models slightly decreasing, but with an original score of 0.97 and 0.95 for BW_I and GA_I they are still performing well when ran on the Illumina data. Overall, both Affymetrix and Illumina models performed well given that data was pertaining from different manufacturers and different groups of studies. Particularly stability around viral performance suggests a robustness within the gene lists for classifying viral samples correctly. However, given that bacterial performance change was very comparable to viral, it too suggests a strong ability to classify bacterial samples, even when moving out of the original dataset.

## 4 Discussion

Due to the amount of relevant data, we focused our analysis on studies from two of the largest microarray platforms, Affymetrix and Illumina. Although RNA sequencing data is being currently used to evaluate molecular responses, the number of publicly available human blood infection samples is significantly lower than those of microarrays. The technologies used in this publication utilize very different methods for detecting mRNA sequences with Affymetrix using a 25 bp capture target while Illumina uses a 50 bp capture target with very different detection methods. This creates larger technical differences that cannot be easily removed using mathematical approaches (Barnes *et al.*, 2005). Simpler solutions are more specifically justifiable and allow for greater interpretation, which is the motivation for feature selection amongst models in biological data. We employed two feature selection algorithms using the Random Forest Classifier over our data: BW and GALGO—both essentially cutting the noise and finding the most significant biological variation responsible for predicting disease state. It is unknown without a brute force search whether a truly optimal combination of genes has been found, however both BW and GA approaches converged around a small group of genes located in uncorrelated and functionally separable clusters. Models were found to be strongly enriched for the ISGs. In fact, IFI27 and LY6E (both ISGs) were included in all Affymetrix and Illumina models. IFI27 is involved in various signalling pathways affecting apoptosis (Gytz *et al.*, 2017; Liu *et al.*, 2014;  Rosebeck and Leaman, 2008). Whereas, LY6E belongs to a class of interferon-inducible factors that broadly enhance viral infectivity (Mar *et al.*, 2018). LY6E has also been attributed a diverse set of effects, including attenuating T-cell receptor signalling (Saitoh *et al.*, 1995) and suppressing responsiveness to lipopolysaccharide that stimulate immune responses (Meng and Lowell, 1997). Moreover, IFI27 was shown by Tang et al. to be a single–gene biomarker that discriminates between influenza and other viral and bacterial infections in patients with suspected respiratory infection (Tang *et al.*, 2017). However, this single-gene biomarker approach lacks generalizability and robustness when predicting a more varied pathogen set. As we have observed, performance in our meta-analysis was greatly improved by including more genes in our models. While Sweeney *et al.* (2016) employed a more multivariate approach their 7 gene strong model only marginally was able to discriminate between bacterial and viral classes in our dataset (Section S5, Supplementary Material). More specifically, we asked the question whether the resulting score was able to discriminate between bacterial, control and viral samples and found that while on average these 7 genes discriminated between viral and bacterial samples a technology dependent threshold is required to optimally separate the classes (Figs S24 and S25, Supplementary Material). Moreover, control samples generally scored similarly to bacterial samples. In a secondary attempt we tried to utilize the same RF approach using the 7 genes provided by the authors and found that in all cases specificity in the model was high but sensitivity was significantly lower than the models we have developed (Tables S32–S37). Our larger set of RF selected genes contained numerous examples confirmed by previous studies to be implicated in disease states. For instance, our results coincide with recent meta-analysis, by Andres-Terre *et al.* (2015), looking at transcriptional signatures of infections, specifically in distinguishing influenza from other viral and bacterial infections, which found 127 multi-gene signatures, 27 of which were also present in our representative models (ATF3, BST2, CXCL10, EIF2AK2, HERC5, HERC6, IFI27, IFI44, IFI44L, IFI6, IFIT1, IFIT2, IFIT5, ISG15, JUP, LGALS3BP, LY6E, MRPL44, MTHFD2, MX1, OAS1, OAS2, OAS3, OASL, RSAD2, RTP4, SERPING1, SPATS2L) serving to validate our successful data integration and biological findings (Andres-Terre *et al.*, 2015). Notably amongst these coinciding genes are IFI27 and LY6E, again confirming the validity of our converging feature selection. To better understand the genes selected by our approach we directly compared the 88 genes that were selected on the basis of having a > 5% inclusion rate (Fig. S14, Supplementary Material). Notably between Affymetrix and Illumina data, the direction of change (up or down-regulation) comparing bacterial, control and viral samples was retained with some clear differences in variation for a subset of genes likely due to the technological differences between the platforms. For example, the gene XIST shows high variability in the Affymetrix dataset and a smaller magnitude of variation within the Illumina data but with a consistent change in the medians across the samples (Fig. S14, Supplementary Material). Similarly, IFI27, one of the key genes identified by our and other authors shows similar response mechanics although with a marginally higher level of expression in Illumina datasets. Overall, the responses, regardless of the two technologies tested, are comparable and contribute to the ability to develop a cross-technology predictive model. By inferring the underlying interaction network, we discovered that convergence was not only happening to a set of genes, but also, and more prominently, convergence was focusing around particular groups of functionally similar genes. This gene-group convergence only emerged as part of an in-depth investigation into the driving forces of feature selection from a biological network perspective. When representative members of these uncorrelated gene clusters are taken together, they can form highly predictive gene lists. With the ability to define the host response to viral and bacterial infections, genes of our identified clusters are likely good at approximating key functions important in disease state prediction. Notably, the four functional groups of genes were indicated to be: Type I ISGs, Chemotaxis genes, Apoptotic Processes genes and Inflammatory/Innate Response genes, which were prevalent in every model (both Affymetrix and Illumina). Within this cluster convergence, we found a highly selected group of genes to be ISGs (the most frequent between both Affymetrix and Illumina models). This is no surprise, given Type I Interferons serve as a link between the innate and adaptive immune systems (Tough, 2004) and have a broad range of effects on both innate and adaptive immune cells during infection with viruses, bacteria and parasites (McNab *et al.*, 2015). While ISGs exact function are not fully understood, it appears that our RF models have identified their strong connection to disease state (Hertzog *et al.*, 2003; Kovarik *et al.*, 2016). Whilst convergence was prominent around four functional groups of genes, we also note that both in Affymetrix and Illumina, a more variable set of functional gene groups were used in addition within our gene lists. Hence, there is a degree of variability in gene solutions, and it seems that there is an interchangeable portion of our gene lists in which a number of genes from uncorrelated functional groups of genes can be used to achieve high performance in defining disease state. Finally, we verified our gene lists for generalizability by retraining and evaluating on data from a different manufacturer to which they were discovered in (Affymetrix Gene lists to Illumina and Illumina Gene lists to Affymetrix). It is apparent that all gene lists tend to do better on Affymetrix data, regardless of which set they were discovered on, which suggests that the dataset, not the gene lists, is influencing performance. Hence, we have uncovered the differentiating biological signatures underlying able to define bacterial and viral infections.

## 5 Conclusion

With the high accuracy that our models achieve within these datasets, stratification and treatment options for relevant individuals can be easily improved through the use of such models. To apply this in clinical settings across larger populations, additional development of a cheap diagnostic test, for example, using PCR or Nanostring, would be required. Importantly, the increase in costs associated with such an initial diagnostic test would be significantly offset by more rationale use of antibiotics in clinical settings and could potentially mitigate the increasingly observed antibiotic resistance. To tackle this challenge, we need to establish better diagnostic tools, linked to computational mechanisms, to provide a more comprehensive detection of diseases and associated treatments. Such personalized medicine approaches can only be supported with models such as developed within this publication. As data availability is growing and healthcare is transforming into the digital age, it is conceivable that our model will have a place in supporting clinical decisions at some point in the future.

## Supplementary Material

btab089_Supplementary_Data
