# Supplementary material for: An OMICs-based meta-analysis to support infection state stratification
Source: Bioinformatics. 2021 Feb 9;37(16):2347–55. doi: 10.1093/bioinformatics/btab089 (PMC8388022; doi:10.1093/bioinformatics/btab089)
Supplement: btab089_Supplementary_Data [file btab089_supplementary_data.zip › Appendix_21122020.docx]

# S1 Pre-processing

### Batch correction pipeline. We developed a two-phase batch correction pipeline, which included the combination of various platforms and studies, depicted in Fig S1. Within this process, batch correction was done using the Combat [1] algorithm.


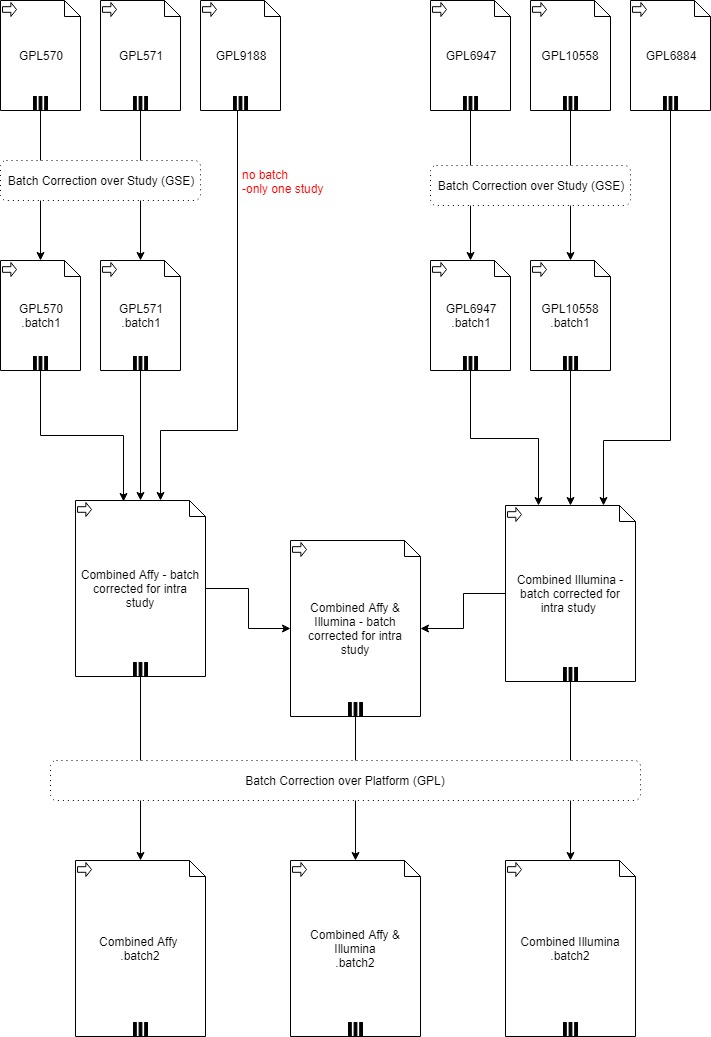


**Fig S1. Batch correction pipeline.** Intra platform study effects are batch corrected first within a platform, then platforms are merged, and batch corrected over platforms, both using sample class as a covariate. Firstly, in each platform, we batch correct over studies (if more than one study is present). We then combined by the intersect of Genes for each manufacturer, initially giving us two combined data sets, one for Affymetrix and one for Illumina. With a final merge we created a fully combined data set. On each combined set, there was technical variation due to platform differences, which we removed with a second round of batch correction.

### Batch correction validation. Results of batch correction were investigated for impact on the underlying biology by comparing dataset differentially expressed genes before and after batch correction using a Fisher's exact t-test [2] and a Hyper Geometric Test [3]. For a significant result, the overlap of differentially expressed genes was significant, and we inferred batch correction had not removed biological variation. Conversely, for a non-significant differentially expressed gene overlap, batch correction had likely removed biological variation and damaged the data quality. Affymetrix platforms were successfully merged and combined via our batch correction pipeline, by both study (Table S1) and platform batch corrections (Table S2). For Illumina datasets we found cross platform merging was not possible without significant change in DE genes. However, in a single platform, GPL10558 we found that studies could be merged, and batch corrected without significant changes in DE genes (Table S1). This platform contained a considerable number of samples (2,432 across 8 studies), more than our Affymetrix combined dataset (Table S4). Hence, we proceeded to use the single platform GPL10558 data instead of creating a combined Illumina dataset. We also attempted to merge both Affymetrix and Illumina datasets (as depicted in Fig S1), however, this resulted in loss of biological variation (data not shown).

**Table S1. Results of Fisher & Hyper Geometric test for changes in gene expression over study level batch corrections of Affymetrix and Illumina studies.** Studies belong to a single platform, which pertains to a single manufacturer. More specifically, for each study we calculated the differentially expressed genes using a simple ANOVA and extracted genes significantly associated with class for the pre-batch and post-batch corrected datasets. For each dataset two gene lists were retrieved at FDR < 10%. Next, we compare whether the overlap between these two lists is significant given the identified genes within the lists using a Fisher’s test and a Hyper Geometric Test. In this table we show the p-values returned by these procedure. A significant p-value would indicate that the overlap is significant and that batch correction has not changed the studies differentially expressed genes, whereas a non-significant (NS) p-value would indicate batch correction has changed differentially expressed genes and impacted the underlying study biology.

| **Manufacturer** | **Platform** | **Study (GSE)** | **Fisher Test p-values** | **Hyper Geometric Test p-values** |
| --- | --- | --- | --- | --- |
| Affymetrix | GPL570 | GSE18090 | 5.18E-14 | 0.00E+00 |
|  | GPL570 | GSE25504 | NS | 0.00E+00 |
|  | GPL570 | GSE28750 | 0.00E+00 | 0.00E+00 |
|  | GPL570 | GSE34205 | 0.00E+00 | 0.00E+00 |
|  | GPL570 | GSE49954 | 2.82E-25 | 0.00E+00 |
|  | GPL570 | GSE50628 | 2.54E-175 | 0.00E+00 |
|  | GPL570 | GSE54992 | 0.00E+00 | 0.00E+00 |
|  | GPL570 | GSE6269 | 0.00E+00 | 0.00E+00 |
|  | GPL570 | GSE66099 | 0.00E+00 | 0.00E+00 |
|  | GPL570 | GSE69606 | 0.00E+00 | 0.00E+00 |
|  | GPL571 | GSE17156 | 0.00E+00 | 0.00E+00 |
|  | GPL571 | GSE52428 | 0.00E+00 | 0.00E+00 |
|  | GPL571 | GSE95104 | 9.91E-42 | 0.00E+00 |
| Illumina | GPL10558 | GSE29385 | 0.00E+00 | 0.00E+00 |
|  | GPL10558 | GSE37250 | 0.00E+00 | 0.00E+00 |
|  | GPL10558 | GSE60244 | 3.90E-23 | 0.00E+00 |
|  | GPL10558 | GSE64456 | 0.00E+00 | 0.00E+00 |
|  | GPL10558 | GSE644562 | 0.00E+00 | 0.00E+00 |
|  | GPL10558 | GSE68310 | 2.96E-23 | 0.00E+00 |

**Table S2. Results of Fisher & Hyper Geometric test for changes in gene expression over platform level batch corrections of Affymetrix datasets.** Batch corrected studies form platform level datasets (for Affymetrix) which are subsequently batch corrected again to remove platform level batch effects. The batch correction was again tested for change in differentially expressed genes before and after batch correction using a Fisher's test and a Hyper Geometric Test. A significant p-value would indicate batch correction has not changed the studies differentially expressed genes, whereas a non-significant (NS) p-value would indicate batch correction has changed differentially expressed genes and impacted the underlying study biology.

| **Manufacturer** | **Platform** | **Fisher Test p-values** | **Hyper Geometric Test p-values** |
| --- | --- | --- | --- |
| Affymetrix | GPL570 | 0.00E+00 | 0.00E+00 |
|  | GPL571 | 0.00E+00 | 0.00E+00 |
|  | GPL9188 | 0.00E+00 | 0.00E+00 |

We also validated that the batch correction removed the previously observed clustering by study, by visualising before and after principle component plots. For Affymetrix data, we display the final PCA [4] after batch correcting over platform (Fig S2), whereas for Illumina, since we are only looking at a single platform, we show the results of batch correction over only studies in Fig S3.


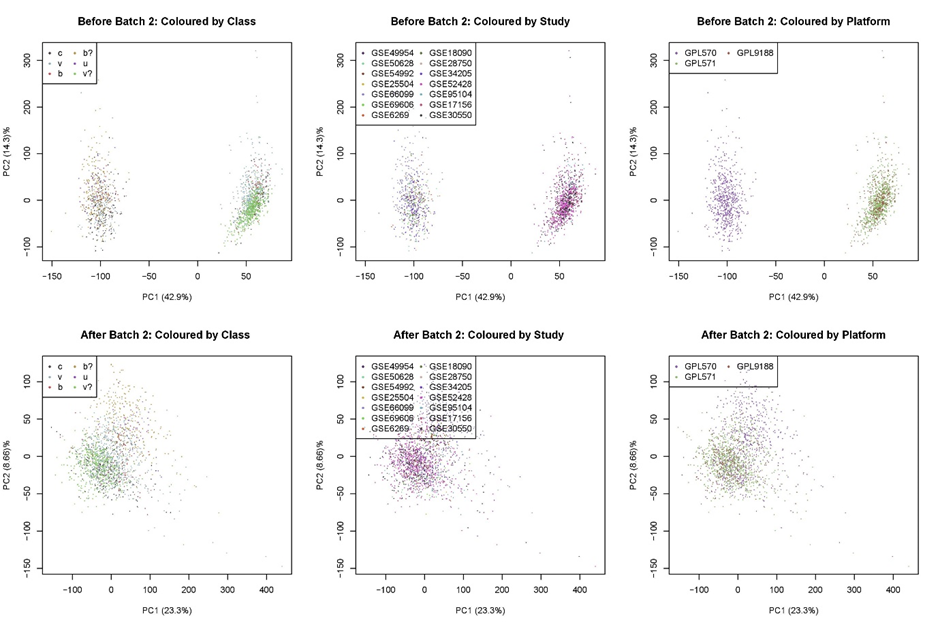


**Fig S2. PCA of before and after batch correction over Study on Affymetrix combined dataset using ComBat.**


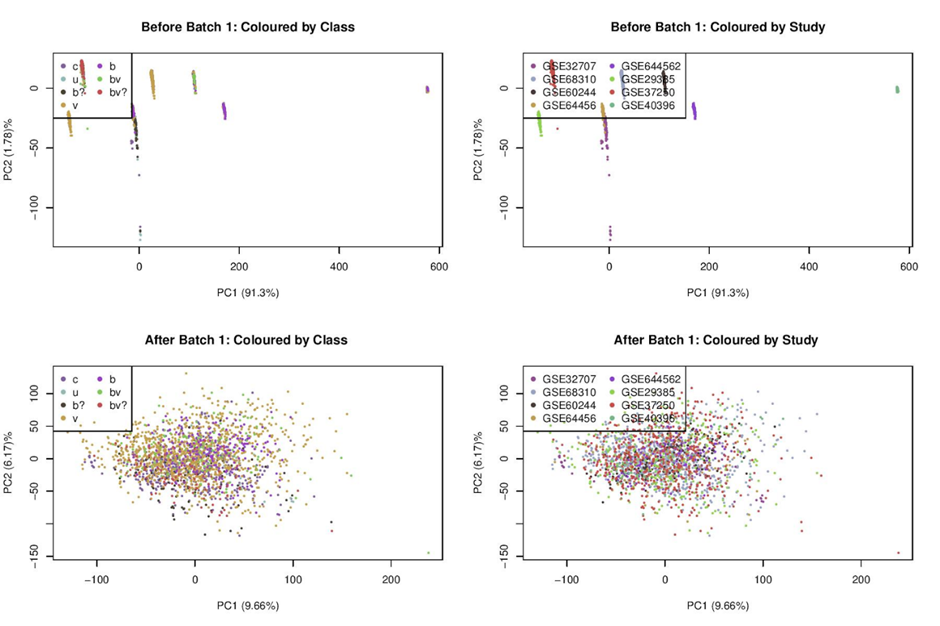


**Fig S3. PCA of before and after batch correction over Study on GPL10885 using ComBat.**

The original GEO records contained several samples labelled with uncertain classifications, which represents lower levels of detection in original study. To reduce this class (classes explained in Table S3) variation present in datasets, we created a confirmed class only dataset removing “b?”, “v?”, “bv?” and “u” samples from the data, for each manufacturer: creating Affymetrix only containing confirmed classes (Affy_C) and Illumina only containing confirmed classes (Illumina_C). Whilst this ‘confirmed class’ dataset may contain samples with more certainty, a problem is that it also removes information which can be used to train models. We also formed a second ‘ambiguous class integrated’ dataset for each manufacturer. This produced a larger version of each dataset, and whilst more varied, provides a means of cross comparison of results. This integrated “v?” to “v” and “b?” to “b”; creating Affymetrix with ambiguous classes integrated (Affy_I) and Illumina with ambiguous classes integrated (Illumina_I).

**Table S3. Sample labelling explanation.**

| Sample Label | Meaning |
| --- | --- |
| b | Bacterial |
| v | Viral |
| c | Control |
| u | Unknown |
| “Sample Label” + ? | Representing uncertainty from lower levels of detection in original study. |

Batch corrected Affymetrix and Illumina data formed further two instances of their respective dataset when splitting by uncertainty: one class confirmed and one ambiguous class integrated instance. For Affymetrix this formed Affy_C and Affy_I of 982 and 1676 samples respectively (Table S4). Whilst for Illumina this formed Illumina_C and Illumina_I of 1751 and 1892 samples respectively. It is evident there is an uneven class distribution present in all 4 datasets. All Affy_C, Affy_I, Illumina_C, and Illumina_I are made up of more than 50% viral samples (66.60%,66.89%,61.05%, and 56.50% respectively)(Table S2). The most under represented class is bacterial samples, especially in the confirmed instances of the data, being 8.86% in Affy_C, and 12.28% in Illumina_C (Table S4). Including the ambiguous samples reduced the bacterial underrepresentation, but still bacterial samples remain a small portion of samples in Affy_I and Illumina_I at 18.74% and 18.82% respectively.

**Table S4 Modelling dataset breakdown.**

| Dataset | Description | Platforms | Bacterial Samples | Viral Samples | Control Samples | Total Samples |
| --- | --- | --- | --- | --- | --- | --- |
| Affy_C | Affymetrix dataset with only confirmed samples | GPL570  GPL571  GPL9188 | 87  (8.86%) | 654 (66.60%) | 241 (24.54%) | 982 |
| Affy_I | Affymetrix dataset with integrated samples | GPL570  GPL571  GPL9188 | 314 (18.74%) | 1121 (66.89%) | 241 (14.38%) | 1676 |
| Illumina_C | Illumina dataset with only samples | GPL10558 | 215 (12.28%) | 1069 (61.05%) | 467 (26.67%) | 1751 |
| Illumina_I | Illumina dataset with integrated samples | GPL10558 | 356 (18.82%) | 1069 (56.50%) | 467  (24.68%) | 1892 |

This table includes the expanded study datasets including the confirmed instances (Affy_C and Ilumina_C) omitted from the main reporting.

# S2 Biomarker search results

## Galgo search procedure convergence

For the GA search procedure, we observed an approach to convergence in evolved chromosomes after 200 generations (Fig S4). To encompass uncertainty around this generation number we ran all search procedures over 250 generations.


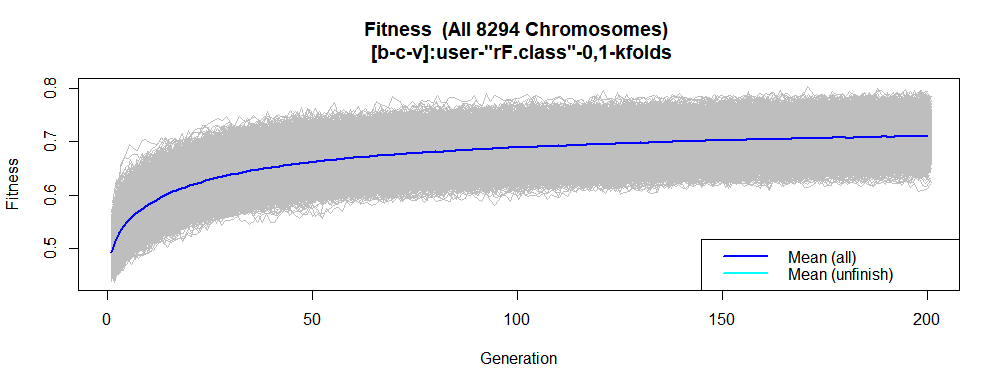


**Fig S4. Example GA run.** Figure shows GA approaching convergence at 200 generations.

## Representative model summary performance

For each dataset we obtained two representative RF models: one generated through BW, and another with GA (Table S5). Model performance was recorded as the size of the gene list and its class-based performance in terms of: Balanced Accuracy, Sensitivity, Specificity, and McNamara’s Test p-value (all derived from the evaluation data split). Average model size was relatively similar between Affymetrix (37.75) and Illumina models (33), however on average Illumina models were smaller. In the proceeding sections each approach is broken down by manufacturer for a more detailed analysis.

**Table S5. Overall optimal model performance.** Broken down by Affymetrix and Illumina data sets, then by feature selection procedure/data instance combinations. C representing the confirmed data instance and I representing the integrated instance.

|  | Model- Dataset Combination | Gene-set Size | Balanced Accuracy (B/C/V) | Sensitivity (B/C/V) | Specificity (B/C/V) | Mcnemar’s Test p-value |
| --- | --- | --- | --- | --- | --- | --- |
| Affymetrix  Identified Models | BW_C | 45 | 0.73 / 0.82 / 0.83 | 0.47 / 0.67 / 0.69 | 0.98 / 0.97 / 0.69 | 0.006645 |
|  | BW_I | 33 | 0.94 / 0.78 / 0.86 | 0.90 / 0.57 / 0.97 | 0.93 / 0.96 / 0.76 | 0.00357 |
|  | GA_C | 37 | 0.84 / 0.85 / 0.86 | 0.69 / 0.72 / 0.98 | 0.99 / 0.98 / 0.74 | 5.4e-12 |
|  | GA_I | 36 | 0.93 / 0.82 / 0.89 | 0.88 / 0.66 / 0.97 | 0.99 / 0.97 / 0.81 | 4.9e-10 |
| Illumina  Identified Models | BW_C | 37 | 0.72 / 0.74 / 0.75 | 0.45 / 0.60 / 0.88 | 0.98 / 0.65 / 0.77 | 0.00038 |
|  | BW_I | 30 | 0.86 / 0.70 / 0.78 | 0.80 / 0.47 / 0.87 | 0.93 / 0.92 / 0.87 | 0.002357 |
|  | GA_C | 28 | 0.52 / 0.51 / 0.91 | 0.52 / 0.51 / 0.91 | 0.98 / 0.93 / 0.56 | 2.2e-16 |
|  | GA_I | 37 | 0.82 / 0.58/ 0.89 | 0.83 / 0.58 / 0.89 | 0.93 / 0.94 / 0.77 | 4.333e-15 |
|  | Average | 35.38 | 0.80 / 0.73 / 0.85 | 0.70 / 0.60 / 0.90 | 0.97 / 0.92 / 0.75 | 0.001619 |

## Backwards elimination represented models

### Affymetrix. After evaluating the robust gene list after backwards elimination on Affy_C and Affy_I, we took forward best-performing models and ran each on the evaluation set. We found that the 45 BW_C and 33 BW_I gene list models performed varying to class (Table S5). The integrated model performed better on Bacterial classes, the smallest class in the dataset. The integrated model also performed particularly well in balanced accuracy (0.94 2dp), which incorporates the bias towards uneven class distribution (Table S5).

While BW_C had the highest specificity amongst classes (0.98 2dp), meaning that 98% of samples classed as not bacterial, were not bacterial. It simultaneously had the lowest sensitivity (0.47 2dp), meaning that only 47% of samples classed as bacterial, were bacterial (Table S5).

In terms of viral performance (the largest class for both data instances), we found a marginal difference in optimal models by including ambiguous samples (Table S5). Both increases in balanced accuracy and specificity were less than 0.07 and could even be explained by variance in results. However, there was a significant increase in sensitivity (from 0.69 to 0.97 2dp) and a similar pattern for bacterial with a large performance increase in sensitivity, nearly doubling to 0.90 2dp. These results together indicate that the inclusion of samples with ambiguous code generally improved performance.

**Table S6 Affymetrix gene lists from backward elimination feature selection.** Two gene lists were derived (A) BW_C from the confirmed classes only and (B) from ambiguous classes integrated. 13 genes were found in common, highlighted in bold.

| Affymetrix |  |  |  |  |  |
| --- | --- | --- | --- | --- | --- |
| BW_C |  |  | **BW_I** |  |  |
| *ALOX5AP* | *FGF9* | ***MS4A4A*** | *ANO10* | ***IFI27*** | *OLAH* |
| *ANXA3* | ***HBA1;HBA2*** | ***MTHFD2*** | *ATP5C1* | ***IFI6*** | ***OTOF*** |
| *AOC3* | *HBB* | *NPL* | *CD177* | *IRAK3* | ***PGD*** |
| *ATF3* | *HERC6* | ***OTOF*** | *CEP55* | *ISG15* | *PSMA6;KIAA0391* |
| *ATP6V1A* | ***IFI27*** | ***PGD*** | ***CXCL10*** | *ITGAM* | *RFTN1* |
| *BCL2A1* | *IFI44* | *PLSCR1* | *DLGAP5* | *KMT5B* | ***RSL24D1*** |
| *CCL2* | ***IFI6*** | *POU3F1* | ***FCER1A*** | ***LY6E*** | *SEPT4* |
| *CD1C* | *IL16* | *RPS4Y1* | *GRB10* | ***MMP8*** | *SULT1B1* |
| *CXCL10* | *IL17RA* | ***RSL24D1*** | *GYG1* | ***MS4A4A*** | ***SYNJ2BP;SYNJ2BP-COX16*** |
| *CYP27A1* | *KLHL36* | *SIGLEC1* | ***HBA1;HBA2*** | ***MTHFD2*** | *TFEC* |
| *EIF4B* | *KLRB1* | *SMO* | *HP* | *NSUN7* | *TSPO* |
| *EXOSC9* | *LAMP3* | ***SYNJ2BP;SYNJ2BP-COX16*** |  |  |  |
| *FCER1A* | ***LY6E*** | *USP18* |  |  |  |
| *FCGR1A;FCGR1B;FCGR1CP* | *MECP2* | *WNT10B* |  |  |  |
| *FCGR1B* | ***MMP8*** | *WWC3* |  |  |  |

**Table S7. Performance metrics for BW-C and BW-I on the Affymetrix Datasets**

|  | Gene-set Size | Balanced Accuracy (B/C/V) | Sensitivity  (B/C/V) | Specificity (B/C/V) | Mcnemar’s Test p-value |
| --- | --- | --- | --- | --- | --- |
| BW_C | 45 | 0.73 / 0.82 / 0.83 | 0.47 / 0.67 / 0.69 | 0.98 / 0.97 / 0.69 | 0.006645 |
| BW_I | 33 | 0.94 / 0.78 / 0.86 | 0.90 / 0.57 / 0.97 | 0.93 / 0.96 / 0.76 | 0.00357 |

**Table S8. Affymetrix BW-C confusion matrix**

| BW_C | | Reference | | |
| --- | --- | --- | --- | --- |
|  |  | Bacterial | Control | Viral |
| Prediction | Bacterial | 7 | 1 | 2 |
|  | Control | 3 | 33 | 2 |
|  | Viral | 5 | 15 | 127 |

**Table S9. Affymetrix BW-I confusion matrix**

| BW_I | | Reference | | |
| --- | --- | --- | --- | --- |
|  |  | Bacterial | Control | Viral |
| Prediction | Bacterial | 55 | 1 | 3 |
|  | Control | 0 | 29 | 4 |
|  | Viral | 6 | 21 | 216 |

For better understanding of the underlying biology discovered by models, we performed functional analysis of the gene lists using DAVID [5] (Fig S5 and Fig S6). This shows in the confirmed class the most enriched and significant term is ‘hsa05144 : Malaria’ which contained two genes of particular interest from BW_C (*KLRB1,* and *CCL2*). *KLRB1* is killer cell lectin-like receptor which, on T and natural killer T cells, functions as a co-stimulatory receptor promoting secretion of *IFNγ* [6] and *CCL2,* a cytokine displaying chemotactic activity and implicated in the pathogenesis of diseases [7]. Malaria is caused by a eukaryotic pathogen, *Plasmodium falciparum*, rather than a virus or bacteria, and there were no samples with eukaryotic pathogen infections (to our knowledge) in our data set. However, it is interesting to note that the genes which have been linked to the “malaria pathway” (or gene set) in KEGG in this case (queried by DAVID), evidently have differential roles in bacterial or viral immune response. Amongst other highly enriched terms with significant p-values are ‘immune response’ and ‘receptor-mediated endocytosis’. For BW_I the most significant term is ‘extracellular exosome’, containing the genes such as *MMP8*, which was common to both lists, suggesting its likely importance for distinguishing disease state.

**Fig S5. DAVID Functional enrichment analysis of genes identified in Affymetrix BW-C.**


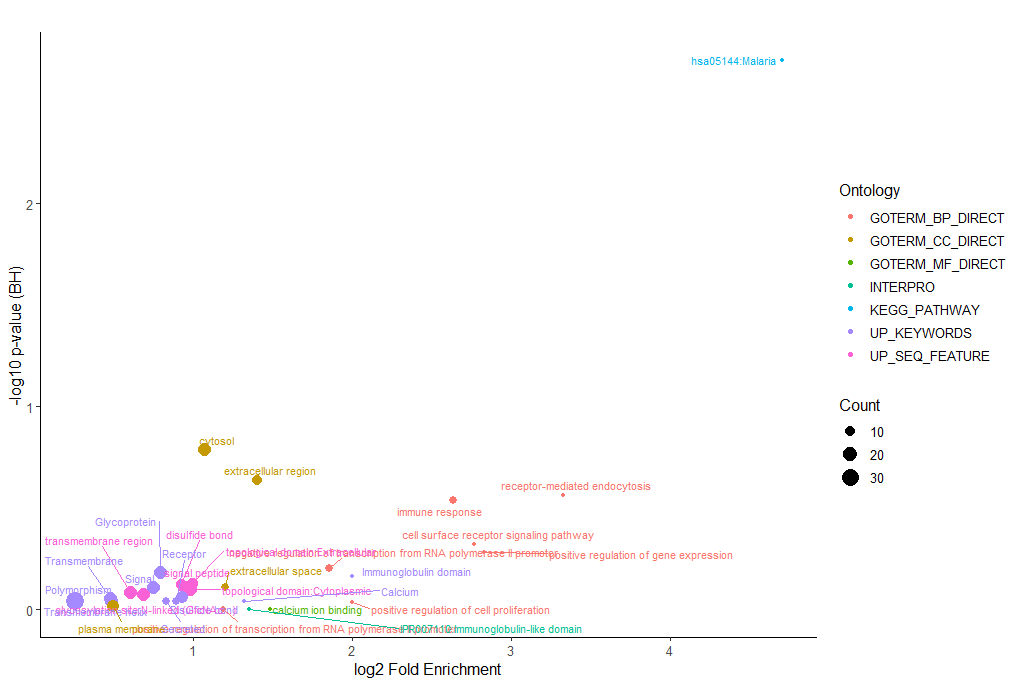

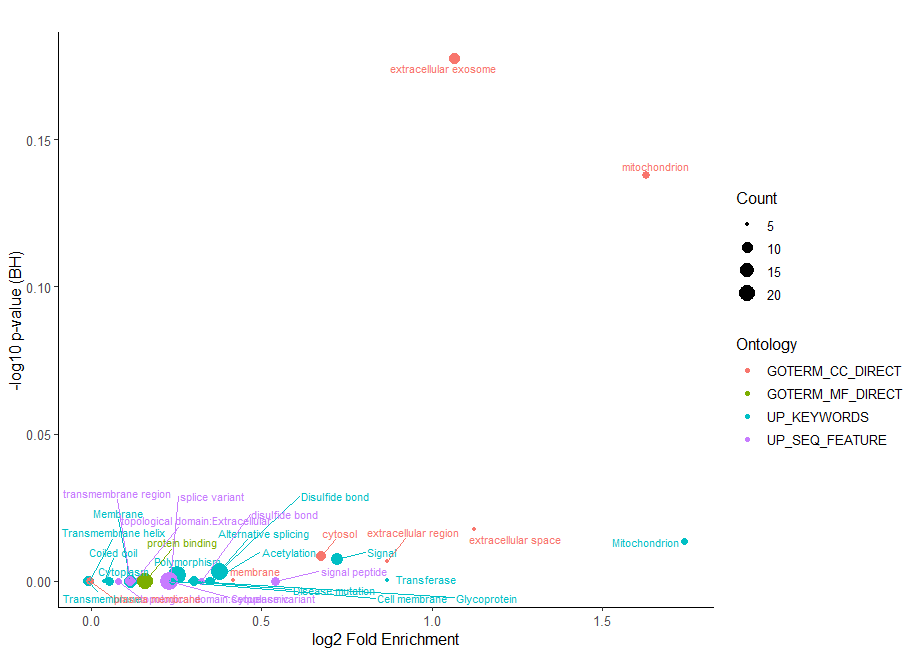


**Fig S6. DAVID Functional enrichment analysis of genes identified in Affymetrix BW-I.**

### Illumina. We next performed similar analysis on the Illumina-data generated models (Table S10). Compared to the Affymetrix models, the Illumina-based models performed less well. The bacterial class exhibited a similar property, being far lower in terms of sensitivity in the confirmed data (0.45 2dp) to the integrated (0.80 2dp) further indicating that a sufficient size of samples is needed for reliable classification.

Interestingly the four genes intersecting Illumina BW_C and BW_I (Table S10) are interferon-induced genes (*IFI27, IFI44, IFI44L,* and *IFIT1*), interferons being a group of signalling proteins made and released by host cells in response to the presence of several viruses [8]. This commonality between the two models is reflected in balanced accuracy, sensitivity, and specificity for the viral class remaining relatively unchanged (Table S11). These interferon-inducible genes, some seen in the prior Affymetrix BW gene lists, are likely to contain significant predictably for viral disease classifications.

**Table S10. Illumina gene lists from backward elimination feature selection.** Two gene lists were derived (A) BW_C from the confirmed classes only and (B) from ambiguous classes integrated. Eight genes were found in common, highlighted in bold.

| Illumina |  |  |  |  |  |
| --- | --- | --- | --- | --- | --- |
| BW_C |  |  | **BW_I** |  |  |
| *ANKRD22* | *GADD45A* | *MPZL1* | *ALKBH5* | *HEATR1* | *PPTC7* |
| *APOBEC3H* | *GYG1* | *MRPL44* | *ARRB1* | ***IFI44*** | ***IFI27*** |
| *ATF3* | *HERC6* | *MYOF* | *CD177* | ***IFI44L*** | *RBM33* |
| *BATF* | *HES4* | *OTOF* | *DSCR3* | ***IFIT1*** | *REPIN1* |
| *BATF2* | *ID3* | *PNKD* | *EEF2* | *IFIT5* | *RETN* |
| *CAMK1D* | ***IFI27*** | *PRC1* | *EIF1* | *INTS8* | *RSAD2* |
| *CCL2* | ***IFI44*** | *PTTG3P* | ***EPSTI1*** | ***LY6E*** | *SAMD9* |
| *CEACAM1* | ***IFI44L*** | *RTP4* | ***FCGR1A;FCGR1CP*** | ***MCEMP1*** | *SLC12A9* |
| *DUSP1* | ***IFIT1*** | *SOCS1* | *GNG2* | *PICALM* | *TSPAN18* |
| *EIF2AK2* | *ISG15* | *SPATS2L* | *GPR84* | *POGK* | *VPS51* |
| *EPSTI1* | *JUP* | *TTPAL* |  |  |  |
| *FCGR1A* | ***LY6E*** |  |  |  |  |
| *FCGR1A;FCGR1CP* | ***MCEMP1*** |  |  |  |  |

**Table S11. Performance metrics for BW-C and BW-I on the Illumina datasets.**

|  | Gene-set Size | Balanced Accuracy (B/C/V) | Sensitivity  (B/C/V) | Specificity (B/C/V) | Mcnemar’s Test p-value |
| --- | --- | --- | --- | --- | --- |
| BW_C | 37 | 0.72 / 0.74 / 0.75 | 0.45 / 0.60 / 0.88 | 0.98 / 0.65 / 0.77 | 0.00038 |
| BW_I | 30 | 0.86 / 0.70 / 0.78 | 0.80 / 0.47 / 0.87 | 0.93 / 0.92 / 0.865 | 0.002357 |

**Table S12. Illumina BW-C confusion matrix**

| BW_C | | Reference | | |
| --- | --- | --- | --- | --- |
|  |  | Bacterial | Control | Viral |
| Prediction | Bacterial | 22 | 2 | 3 |
|  | Control | 7 | 55 | 22 |
|  | Viral | 20 | 35 | 184 |

**Table S13. Illumina BW-I confusion matrix**

| BW_I | | Reference | | |
| --- | --- | --- | --- | --- |
|  |  | Bacterial | Control | Viral |
| Prediction | Bacterial | 58 | 12 | 9 |
|  | Control | 4 | 49 | 18 |
|  | Viral | 11 | 43 | 174 |

Functional analysis of Illumina BW_C in DAVID found the most enriched and significant term ‘Antiviral defence’, followed by ‘defence response to virus’ and ‘Innate immunity’ (Fig S7 and Fig S8). This antiviral presence in the gene lists is unsurprising as the confirmed instance of the data contained such a small set of control and bacterial samples. In the integrated instance, the most enriched and significant term is ‘poly(A) RNA binding’. The poly(A) RNA family of binding proteins contains a number of genes which contribute to the fine-tuning of gene expression in tissue-specific disease [9]. This indicates that several genes in this this family, like the interferon family of genes also present in the list, are highly predictive when used together for diagnosing disease state. While antiviral terms are not seen in functional analysis of Illumina BW_I, it is likely that the remaining anti-viral genes are still present, as they share overlap, particularly over the interferon type I family of inducible genes (*IFI27, IFI44, IFI44L, IFIT1, LY6E*).

**Fig S7. DAVID Functional enrichment analysis of genes identified in Illumina BW-C**


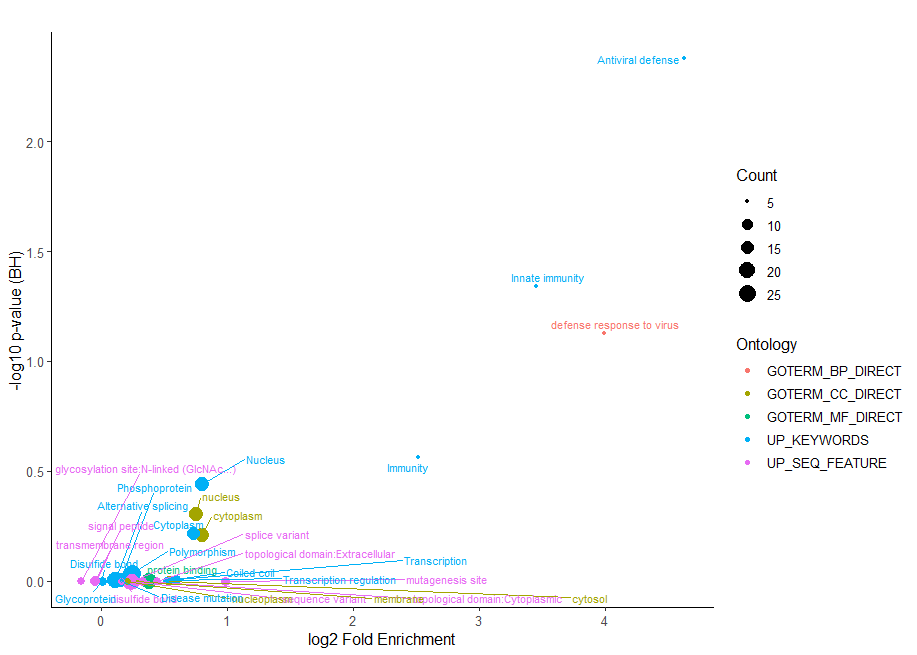

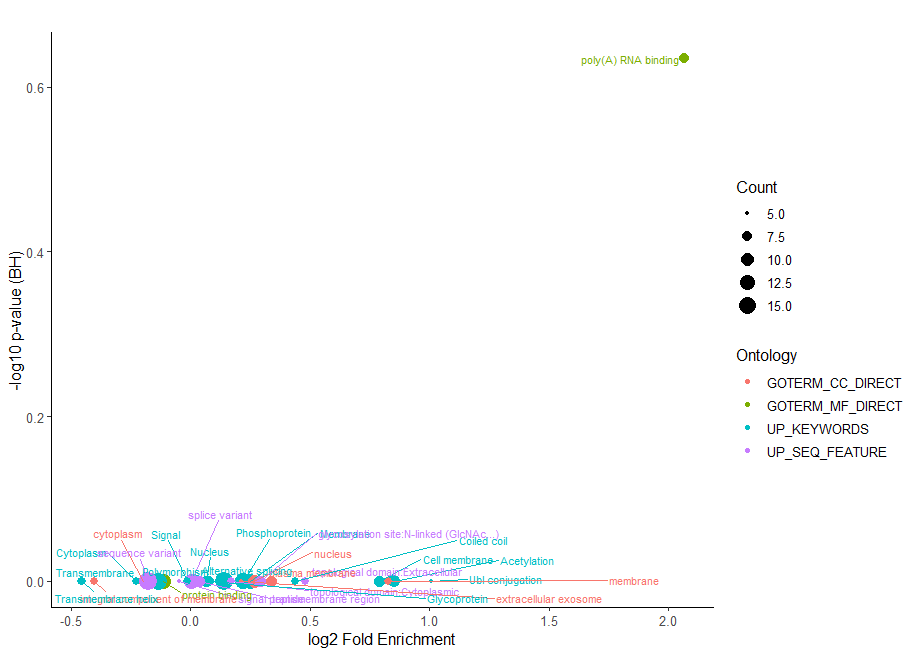


**Fig S8. DAVID Functional enrichment analysis of genes identified in Illumina BW-I.**

## Genetic-algorithm representative models

Each dataset (Affy_C, Affy_I, Illumina_C, and Illumina_I) was run with the same parameters and the same classifier over 250 generations. Similarly to backwards elimination we constructed a representative model for each search procedure ran on a given dataset (in the case of GA this was constructed by using a forward selection strategy over the evolved chromosome set).

### Affymetrix. Table S14 shows the genes in the representative model chosen, using the forward selection strategy, over the two Affymetrix datasets. Out of sample performance for each model, is also reported in Table S15. When considering accuracy alone, it may seem that the smaller model on the ambiguous class integrated dataset (GA-I) performed marginally better, the smaller Mcnemar’s Test p-value indicates that the larger 37 gene GA-C, in fact, performed marginally better (Table S15). In both models, 14 genes are shared, showing that even when including ambiguity amongst samples, there remains a degree of convergence to common genes between the predictive models. While it may look like the fully combined dataset performs marginally better, Mcnemar’s Test p-value shows that the model developed on the slightly smaller dataset performs marginally better.

**Table S14. Affymetrix gene lists from Genetic-algorithm optimized feature selection.** Two gene lists were derived (A) GA-C from the confirmed classes only and (B) from ambiguous classes integrated, GA-I. Out of the 36/37 genes in each model 14 are shared between the two models, showing that the approach has converged to use similar genes in the predictive models.

| Affymetrix |  |  |  |  |  |
| --- | --- | --- | --- | --- | --- |
| GA-C |  |  | **GA-I** |  |  |
| *ANXA3* | ***HPR.HP*** | *OAS3* | ***ANXA3*** | *GRB10* | *PCNX2* |
| *ATF3* | ***IFI27*** | ***PGD*** | *ATP5C1* | *GYG1* | ***PGD*** |
| *BCL2A1* | ***IFI44*** | *RSL24D1* | ***BCL2A1*** | ***HBA1.HBA2*** | *RETN* |
| *BST2* | *IFI44L* | *SEPT4* | *CD177* | ***HBB*** | *RGS1* |
| *CCL2* | *IFI6* | *SIGLEC1* | *CD69* | ***HPR.HP*** | *TFEC* |
| *CD1C* | ***ISG15*** | *SPATS2L* | ***CEP55*** | ***IFI27*** | *TFPI2* |
| *CEP55* | *LAMP3* | *TAOK2* | *CHMP5* | ***IFI44*** | *TMCO1* |
| *DAAM2* | *LGALS3BP* | *TPST1* | *CNIH4* | ***ISG15*** | *TSPO* |
| *EEF1D* | ***LY6E*** | ***WNT10B*** | *CXCL10* | ***LY6E*** | ***WNT10B*** |
| *HBA1.HBA2* | ***MMP8*** | *ZMYM6* | *DLGAP5* | ***MMP8*** | *XIST* |
| *HBB* | ***MS4A4A*** | *ZNF473* | *ERH* | ***MS4A4A*** | *RAD51AP1* |
| *HERC6* | *MX1* |  | *FCER1A* | *MTHFD2* | *PSMA6.KIAA0391* |
| *HLA.DRB4* | *NDUFB2* |  |  |  |  |

**Table S15. Model performance metrics of Affymetrix GA-C and GA-I on out of sample.**

|  | Gene-set Size | Balanced Accuracy (B/C/V) | Sensitivity  (B/C/V) | Specificity (B/C/V) | Mcnemar’s Test p-value |
| --- | --- | --- | --- | --- | --- |
| GA_C | 37 | 0.84 / 0.85 / 0.86 | 0.69 / 0.72 / 0.98 | 0.99 / 0.98 / 0.74 | 5.4e-12 |
| GA_I | 36 | 0.93 / 0.82 / 0.89 | 0.88 / 0.66 / 0.97 | 0.99 / 0.97 / 0.81 | 4.9e-10 |

**Table S16. Affymetrix GA-C confusion matrix**

|  | | Reference | | |
| --- | --- | --- | --- | --- |
|  |  | Bacterial | Control | Viral |
| Prediction | Bacterial | 60 | 4 | 2 |
|  | Control | 6 | 173 | 10 |
|  | Viral | 21 | 64 | 642 |

**Table S17. Affymetrix GA-I confusion matrix**

|  | | Reference | | |
| --- | --- | --- | --- | --- |
|  |  | Bacterial | Control | Viral |
| Prediction | Bacterial | 277 | 1 | 11 |
|  | Control | 12 | 160 | 23 |
|  | Viral | 25 | 80 | 1087 |

Functional analysis of the genes identified within the ‘confirmed classes’ dataset revealed ‘type I interferon signalling signature pathway’ terms as both enriched and significant as well as other anti-viral defence mechanisms (Fig S9 and Fig S10). Analysis of the ‘integrated classes’ dataset also showed several significant terms, one of which was “extracellular region” proteins.

**Fig S9. DAVID Functional enrichment analysis of genes identified in Affymetrix GA-C.**


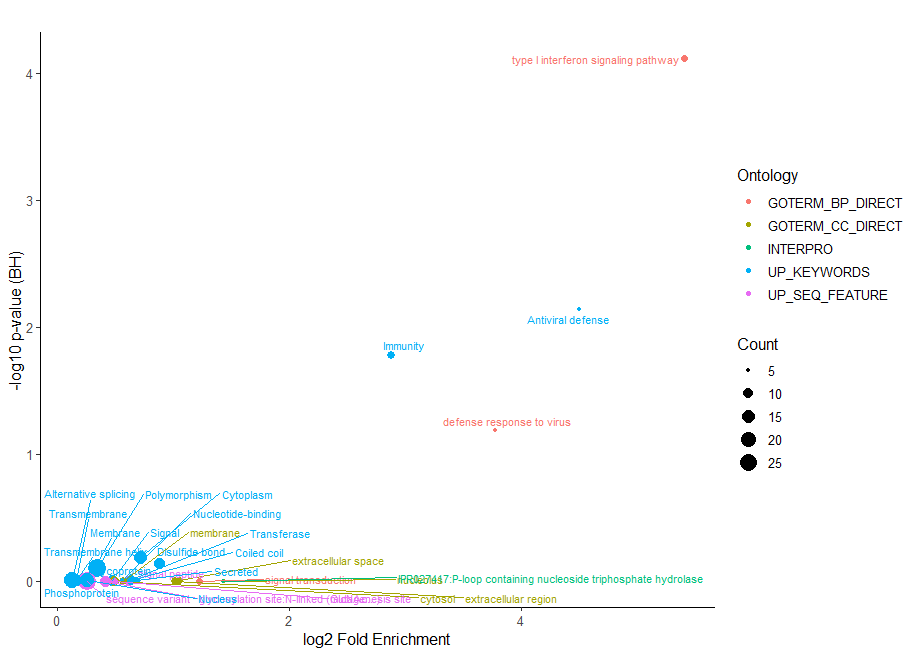

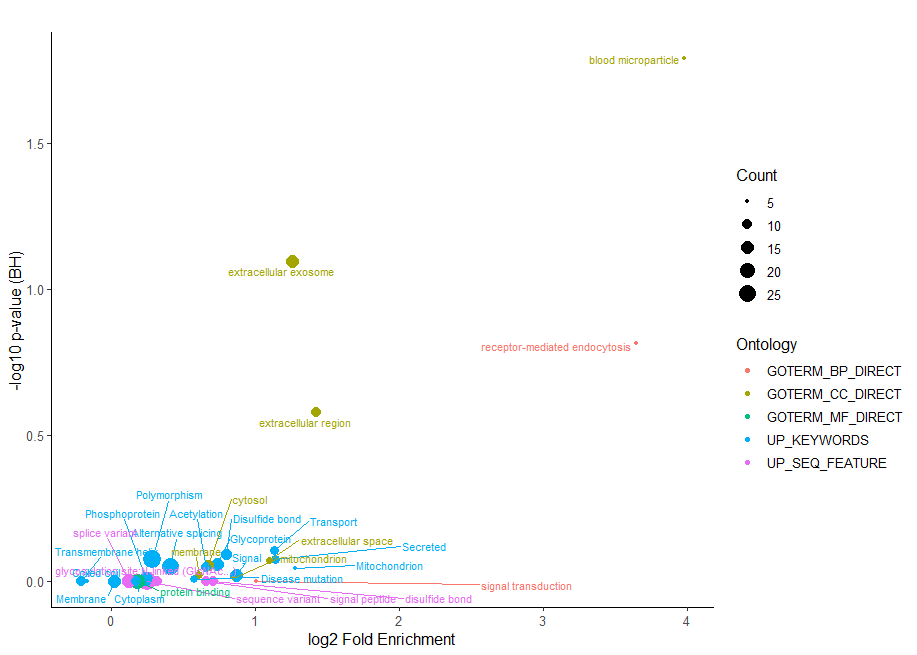


**Fig S10. DAVID Functional enrichment analysis of genes identified in Affymetrix GA-I.**

### Illumina. Table S18 shows the genes included in GA’s representative model on the Illumina_C and Illumina_I data. Particularly interesting is that we see a family of type I interferon-inducible genes being present in combination with a variety of other genes. This would suggest that there likely is a fixed set of functions or a single function that can be used in combination with a non-fixed set enabling accurate prediction of disease state.

**Table S18. Illumina Gene lists from Genetic-algorithm optimized feature selection.** Two gene lists were derived (A) BW_C from the confirmed classes only and (B) from ambiguous classes integrated.

| Illumina |  |  |  |  |  |
| --- | --- | --- | --- | --- | --- |
| GA_C | | | **GA_I** | | |
| *ANKRD22* | *HSH2D* | *MX1* | *AIM2* | ***IFI44*** | ***OASL*** |
| *BNIP2* | ***IFI27*** | *MX2* | *C16orf58* | ***IFI44L*** | ***OTOF*** |
| *CEACAM1* | ***IFI44*** | ***OAS2*** | *CD177* | ***IFIT1*** | *PDCD1* |
| *EIF2AK2* | ***IFI44L*** | ***OAS3*** | *CDCA5* | *IFIT2* | ***PHTF1*** |
| *EPSTI1* | ***IFIT1*** | ***OASL*** | *CDCA7* | *IFIT5* | *PRC1* |
| *FCGR1B* | ***ISG15*** | ***OTOF*** | ***EPSTI1*** | *IFITM3* | *PTTG1* |
| *GYG1* | *JUP* | ***PHTF1*** | *FBXO6* | ***ISG15*** | *RSAD2* |
| *HERC5* | ***LY6E*** | ***SPATS2L*** | *FCGR1A;FCGR1CP* | ***LY6E*** | *SERPING1* |
| *HERC6* | *MAP4K1* |  | *GPR84* | ***MCEMP1*** | ***SPATS2L*** |
| *HES4* | ***MCEMP1*** |  | ***HERC5*** | *MCM4* | *TBK1* |
|  |  |  | *HK3* | *OAS1* | *ZDHHC19* |
|  |  |  | *HPGD* | ***OAS2*** |  |
|  |  |  | ***IFI27*** | ***OAS3*** |  |

Table S19 shows the result of the optimization for the Illumina datasets. These two models show the largest overlap, sharing 15 genes in common (more than half all the genes in GA_C). However, being optimal for the dataset, GA_C on Illumina performed the worst in terms of Bacterial and Control samples. This is likely down to the smaller size of features used to make a prediction. This stands to highlight that even with a small list, reasonable predictions can be achieved, as presented with its high balanced accuracy on viral (0.91 2dp).

**Table S19. Model performance metrics of Illumina GA-C and GA-I**

|  | Gene-set Size | Balanced Accuracy (B/C/V) | Sensitivity  (B/C/V) | Specificity (B/C/V) | Mcnemar’s Test p-value |
| --- | --- | --- | --- | --- | --- |
| GA_C | 28 | 0.52 / 0.51 / 0.91 | 0.52 / 0.51 / 0.91 | 0.98 / 0.93 0.56 | 2.2e-16 |
| GA_I | 37 | 0.82 / 0.58/ 0.89 | 0.83 / 0.58 / 0.89 | 0.93 / 0.94 / 0.77 | 4.333e-15 |

**Table S20. Illumina GA-C confusion matrix**

|  | | Reference | | |
| --- | --- | --- | --- | --- |
|  |  | Bacterial | Control | Viral |
| Prediction | Bacterial | 112 | 11 | 13 |
|  | Control | 13 | 241 | 88 |
|  | Viral | 90 | 215 | 969 |

**Table S21. Illumina GA-I confusion matrix**

|  | | Reference | | |
| --- | --- | --- | --- | --- |
|  |  | Bacterial | Control | Viral |
| Prediction | Bacterial | 294 | 50 | 7 |
|  | Control | 11 | 269 | 94 |
|  | Viral | 51 | 147 | 948 |

We again functionally annotated the identified representative model genes from derived from both GA datasets in Illumina (Fig S11 and Fig S12). In both we found the most significant term to be ‘Antiviral defence’ and most enriched as ‘negative regulation of viral genome replication’. It is also of particular interest again that both analyses found significant and enriched terms related to interferons, in GA_C ‘type I interferon signaling pathway’ which while may seem biased towards viral predictions but has also implicated in varied roles in bacterial infections [10].

**Fig S11. DAVID Functional enrichment analysis of genes identified in Illumina GA-C.**


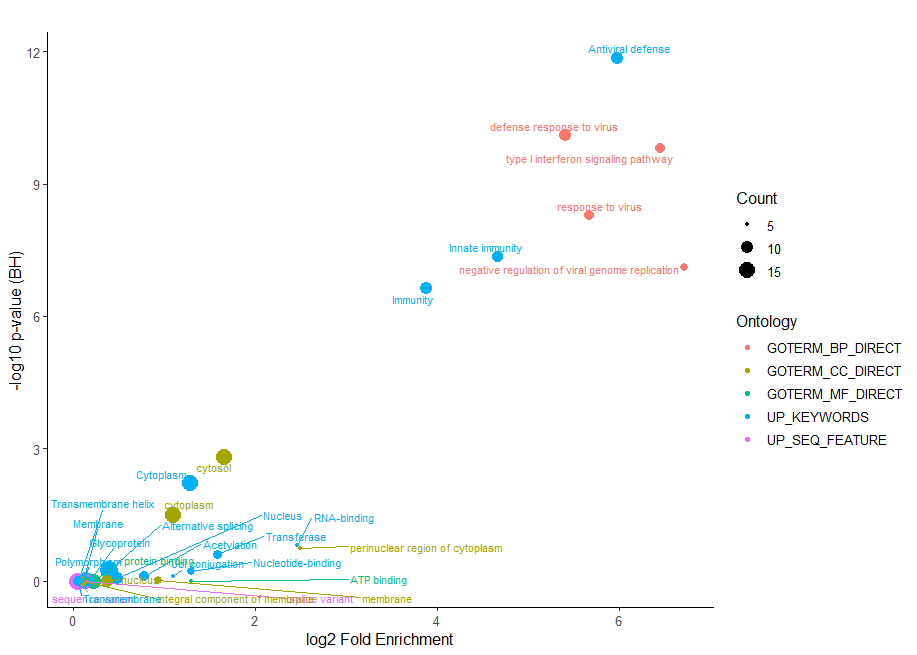

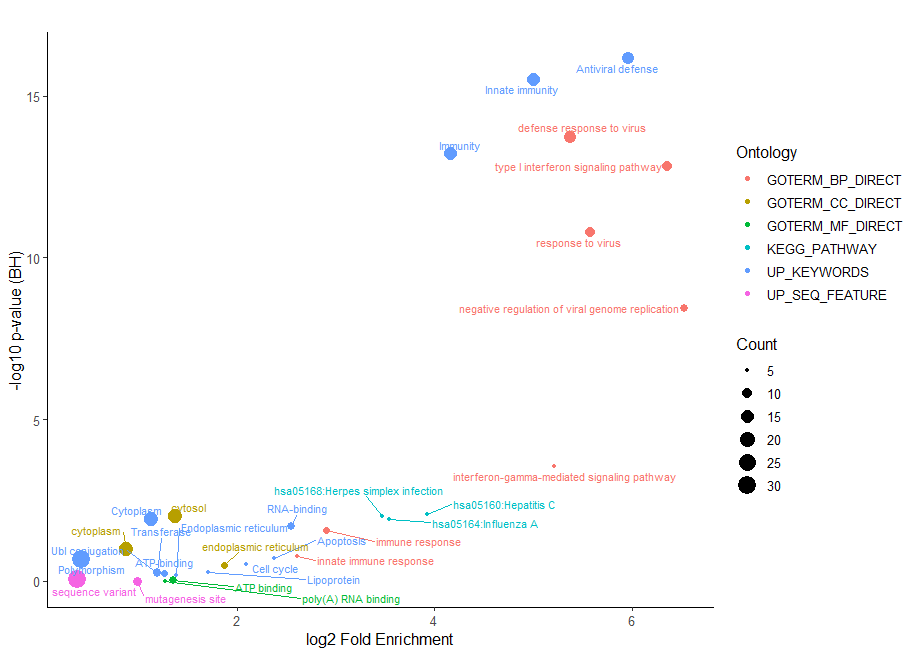


**Fig S12. DAVID Functional enrichment analysis of genes identified in Illumina GA-I.**

# Full feature set model gene importance comparison to frequency of selection in feature selection processes

To explore the benefits of feature selection we compared the top 88 genes indicated to be important between the naïve full models and our feature selection runs (Fig S13). For the full model we took feature importance’s from the models fitted to each dataset (Affymetrix-C,Affymetrix-I, Illumina-C, and Illumina-I) and summed them together. We derived a comparable metric for gene importance from the feature selection process by looking at relative selection frequencies, as in the main section *Identifying biomarker panels predictive of viral, bacterial, and no-infection* which resultant list and ranks can be found in Table S38.

In total we found correspondence with 36/88 intersecting both lists (*FCGR1B, IFIT1, IKZF2, PARP12, OAS3, TMEM123, ANO10, CD1C, MS4A4A, HERC5, OLAH, RETN, SPATS2L, HERC6, SIGLEC1, MX1, PGD, RTP4, SULT1B1, RSAD2, IFIT2, OTOF, NSUN7, MTHFD2, CD177, HK3, IFI44, GRB10, ISG15, HP, MMP8, ITGAM, IFI44L, IFI27, LY6E, TSPO*) (Fig S13). Within this intersection is *IFI27* and *LY6E* two of the genes indicated as important in the manuscript given their intersection of our optimal models. In terms of differences 52 genes (*LMO2, IMPA2, ADAM9, MAFB, IL1R2, XAF1, BMI1;COMMD3-BMI1, MYL12A, UNC45A, DDX60, CD63, LPIN1, SNTB1, TRIM5, DYSF, DNMT1, PLSCR1, CR1, TMCO3, ECI1, TMEM126B, IFIH1, GIMAP6, SP110, FCGR1A;FCGR1B;FCGR1CP, LYRM1, BLVRA, TWISTNB, DLC1, PYGL, KPNA5, ATXN7L3B, IFITM1, IRF7, TBK1, OASL, WDR83OS, RNASE2, CD2, PLIN3, GBP1, PFKFB2, ZBTB5, LTB4R, IL17RA, PHF11, IFI16, SLC12A9, EIF1, OAS1, APOBEC3G, and SOCS3*) found in the full feature model were not found amongst the top genes in our feature selection runs, conversely another 52 top genes (*LGALS3BP, POLR2K, FAM76A, RUNX2, WNT10B, RGS1, TAOK2, ATP5C1, ATF3, PCNX2, LDB1, SMO, DDAH2, TMEM260, MKNK1, PCOLCE2, ADAMTS3, BAZ1B, RSL24D1, LAMP3, HBB, GADD45A, MAN2A2, GCLM, XIST, CHMP5, CCL2, CNIH4, STAMBPL1, IRAK3, CCAR2, SSH1, CDC27, TTK, RPS6KA5, BATF, DLGAP5, RFTN1, SEPT4, BST2, GAPDH, DHX58, ARG1, KIF11, FCER1A, GYG1, IFI6, FCGR1A, PTTG1, SERPING1, OAS2, and IFIT5*) found in our feature selection runs were not found highly important in the full models.


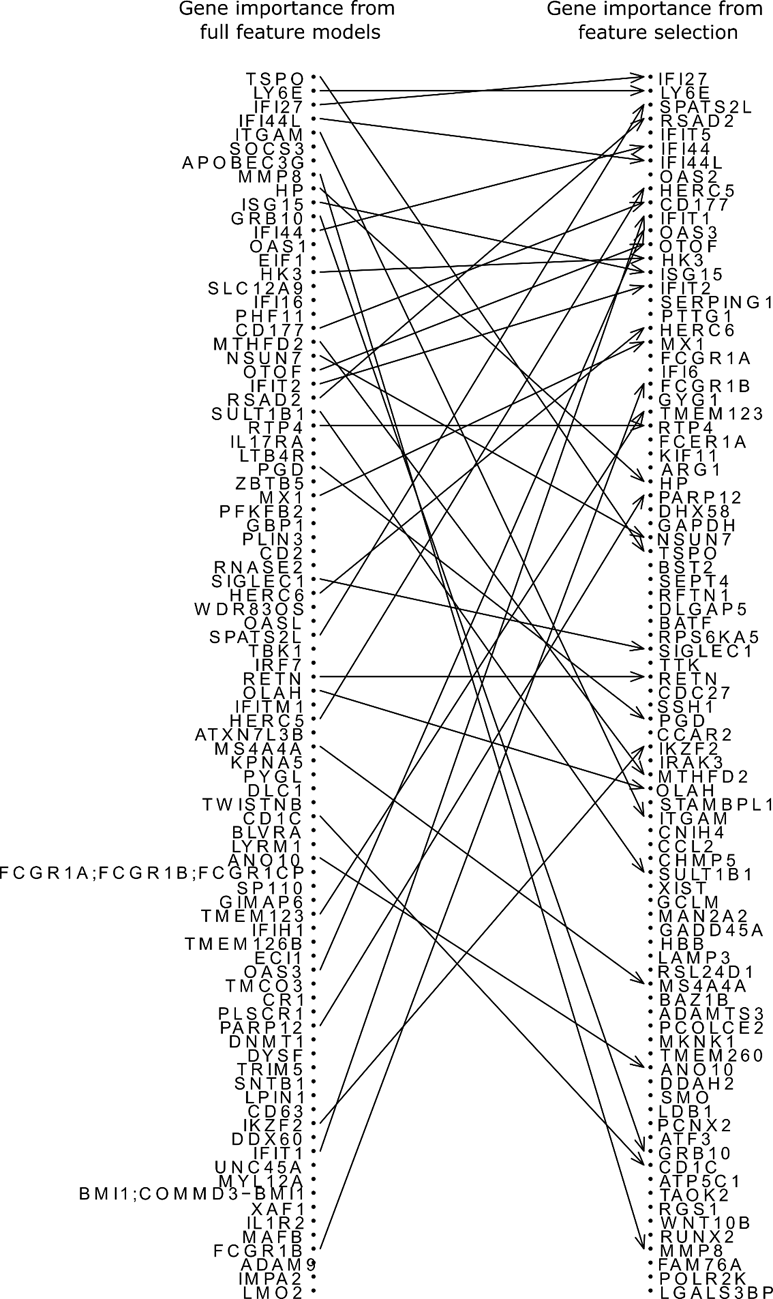


**Fig S13 Correspondence between top ranked 88 genes in full model versus feature selection.** Ranks derived from (i) summed gene importance’s between full feature set models (left), and (ii) gene selection frequency from the backwards BW and GA feature selection runs (right). Corresponding genes are mapped between the two lists.

# Top gene comparison between manufacturer datasets


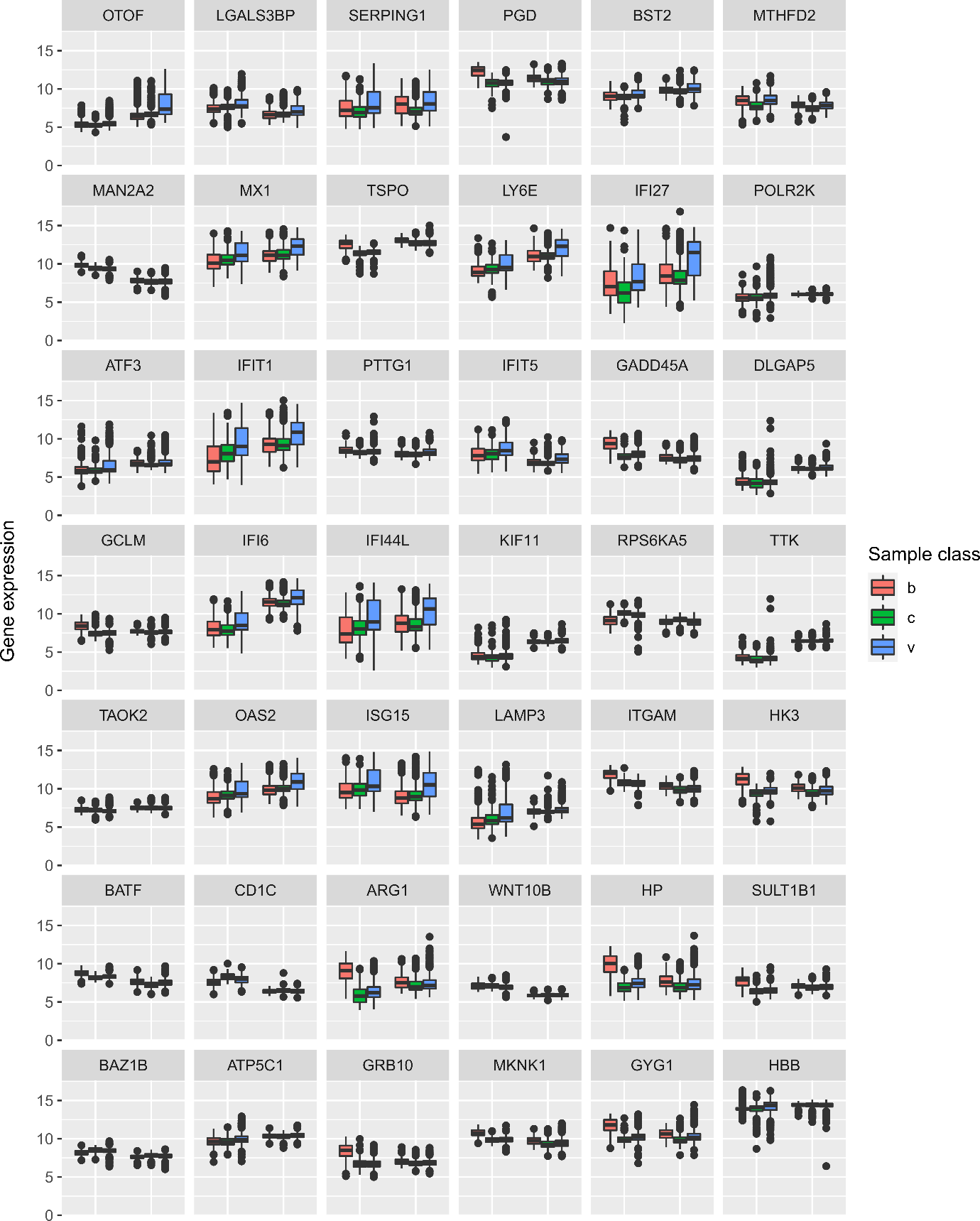
We investigated the expression levels of the 88 genes with >5% inclusion rates (from the feature selection frequency analysis in main manuscript results) between both Affymetrix and Illumina batch corrected data (Fig S14 ).


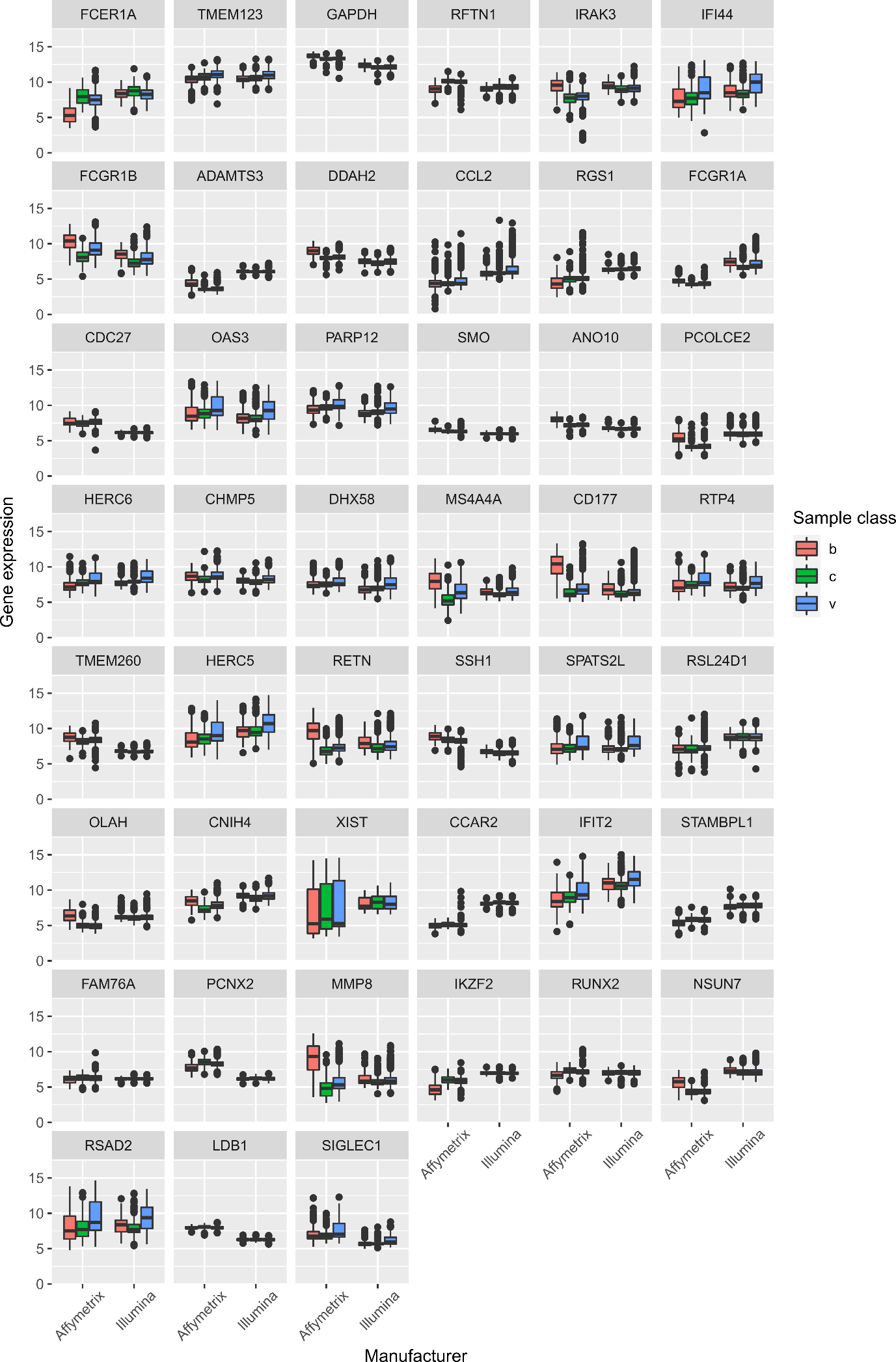


**Fig S14 Boxplots of 88 highly selected gene expressions in Affymetrix and Illumina data by sample classes.**

# S3 Inferred Interaction Networks

We inferred the underlying gene regulatory networks for both Affymetrix and Illumina datasets (visualisation of networks in Fig S26 and S27). GLay clustering of the gene interaction network initially revealed 14 clusters (containing more than 10 genes) in the Illumina network and 16 in the Affymetrix network (Table S22). For gene clusters, either indicated as involving genes “functionally relevant” in the immune response (FR) as indicated by enrichment analysis, or containing genes selected by our models, we further sub clustered them into a maximum depth of three levels of hierarchy. For Illumina data this resulted in 110 distinct groups of genes, and 53 for Affymetrix data. The large difference here is for two reasons (i) the Illumina network contained 6456 more nodes than the Affymetrix Network, and (ii) more refinement was paid to the Illumina interaction network, which was the focus of the rest of cross comparative analysis.

**Table S22. Affymetrix and Illumina Interpreted Inferred Interaction Network Properties.**

| Manufacturer | Nodes (Genes) | Sub-clusters of more than 4 Genes | FR Clusters (% of all) | FR Clusters selected by > 1 Model (% of all) | FR Clusters selected by all four Models (% of all) |
| --- | --- | --- | --- | --- | --- |
| Affymetrix | 13383 | 53 (1.00) | 12 (0.26) | 8 (0.15) | 5 (0.09) |
| Illumina | 19839 | 110 (1.00) | 24 (0.21) | 10 (0.09) | 4 (0.04) |

Clusters have been labelled either functionally related to the immune response (FR). For a cluster to be labelled as FR, functional enrichment analysis of their gene list will have revealed terms both enriched and significant implicated in the host response to disease.

In Affymetrix 12 (26%) of clusters had enriched and significant terms related to functions of the immune system, as indicated by DAVID functional enrichment analysis (Table S22). These 12 FR clusters contained 52 genes out of the union of Affymetrix model genes (64% of all 81 Affymetrix selected genes in the network). Eight of these clusters had been selected by at least one Affymetrix model, and five of these had been selected by all four models.

In Illumina 24 of the 110 clusters had enriched and significant terms related to functions of the immune system in our DAVID analysis (Table S22). Of these 24 FR clusters, 10 had at been selected by at least one Illumina model. These 10 clusters contained 55 genes in the union of Illumina models (68% of all 81 Illumina selected genes in the network). Similar to Affymetrix, a small number of clusters (four) were selected by every model.

For both Affymetrix and Illumina, see the included pdf files showing the clustered network, and the csv files in the supplementary data detailed the exact genes for a number of highly converged to clusters (Figure S26-S27 and Table S39).

## Affymetrix Highly Selected Clusters

We investigated each cluster each FR Clusters selected by all four Models using DAVID enrichment analysis and present the results below in Fig S**16** to S19.

For Cluster 1.1 we found interesting significant terms, namely ‘extracellular exosome’ which had come up previously in the functional enrichment analysis of the Affymetrix Models. Another interesting observation was in Fig S**16**, containing a number of terms related to the immune system, but most interestingly, a number related directly to response to bacteria, ‘antibacterial humoral response’, and ‘phagocyte recognition’.


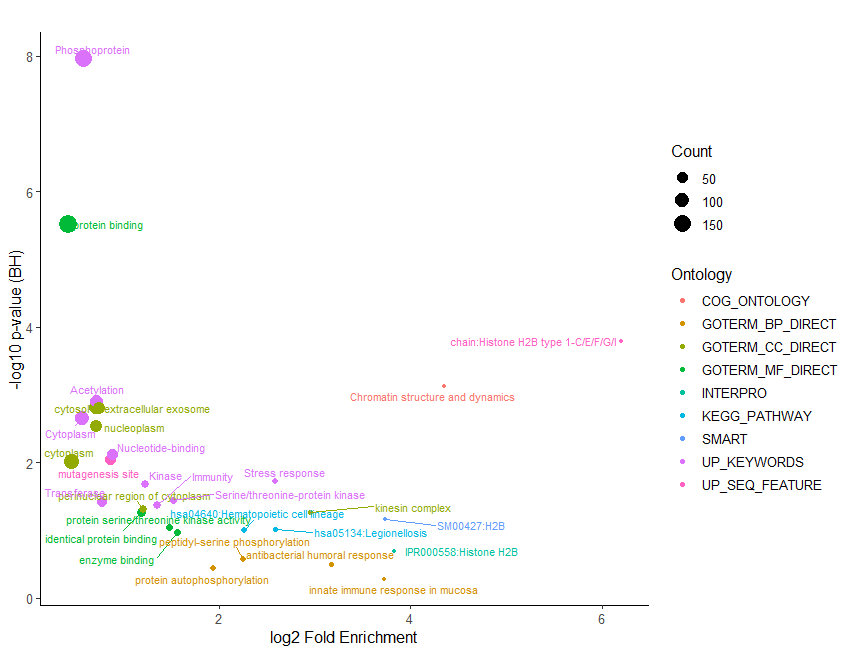


**Fig S15. Results of DAVID functional enrichment analysis for genes in Affymetrix Cluster 1.1.**

For Cluster 1.4 we found the most significant term to be ‘Immunoglobulin C region’, a key component involved with the immune response [11] albeit with an insignificant p-value (Fig S16). In cluster 1.7 we also found a number of terms related to the immune response, including ‘immunity’ and ‘inflammatory response’ (Fig S17). Finally for Clusters 2.3 and 5 we found a number of terms relating to viral infection, namely ‘viral transcription’ in cluster 2.3 (Fig 19), and ‘Antiviral defence’ and ‘Innate Immunity’ (Fig 20).


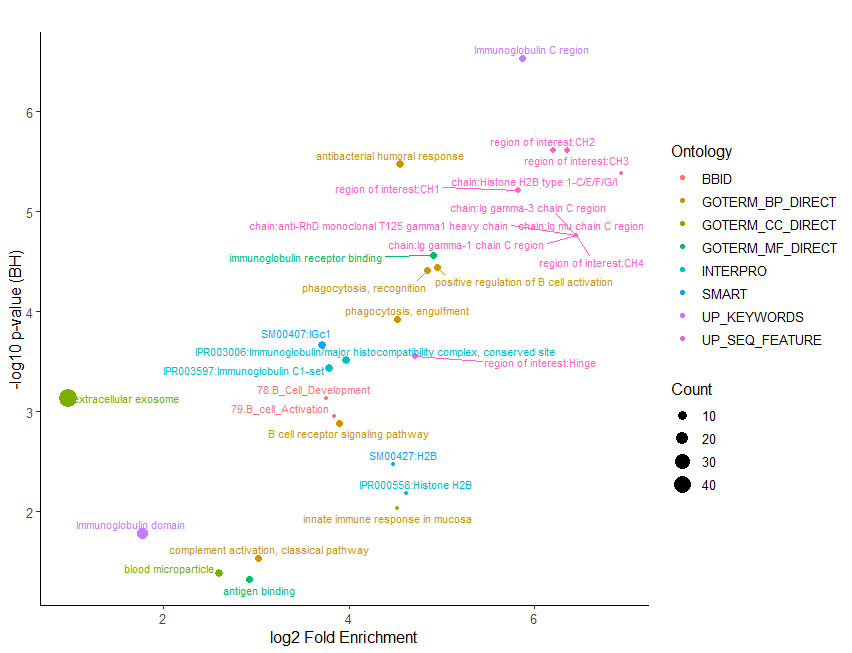


**
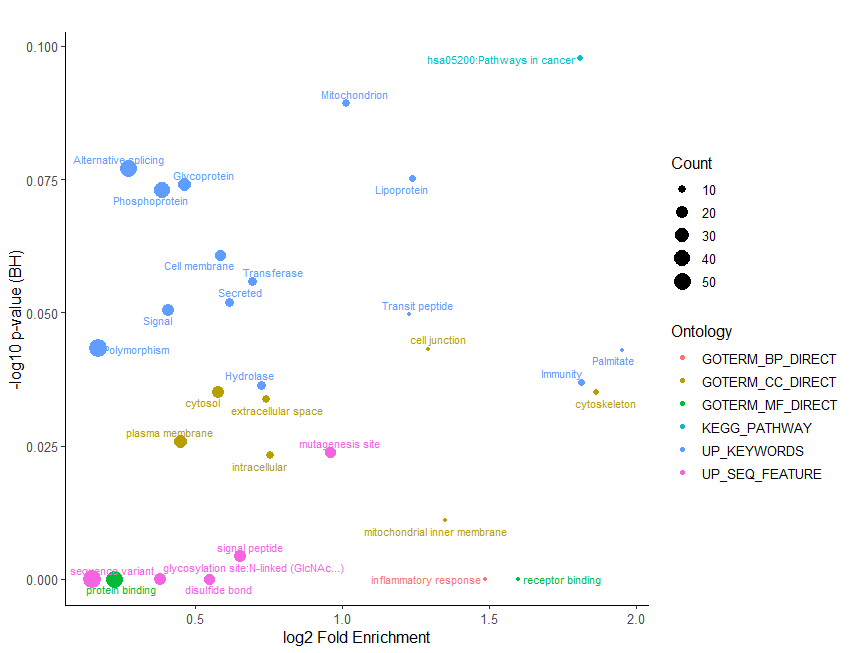
Fig S16. Results of DAVID functional enrichment analysis for genes in Affymetrix Cluster 1.4.**

**Fig S17. Results of DAVID functional enrichment analysis for genes in Affymetrix Cluster 1.7.**

**
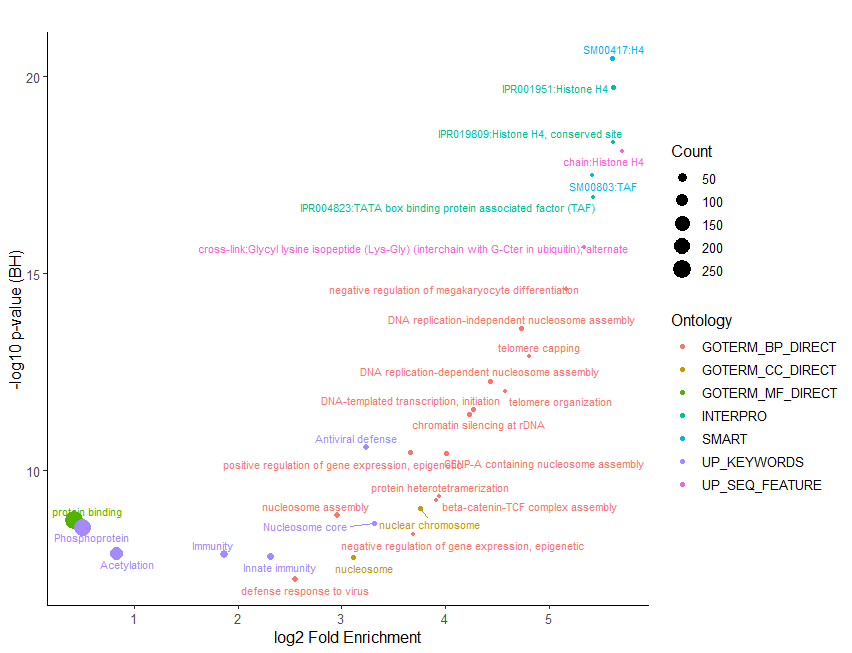
**
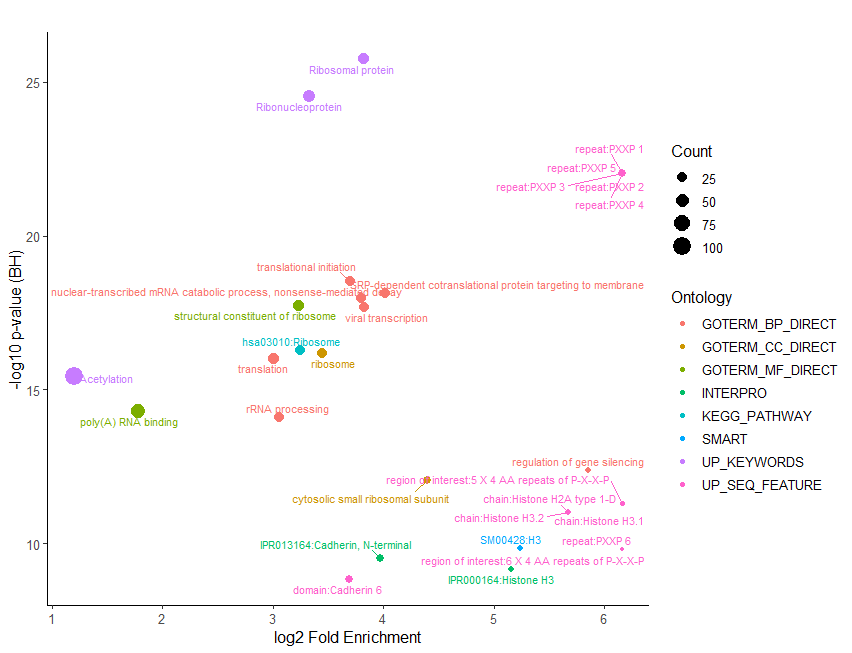


**Fig S18. Results of DAVID functional enrichment analysis for genes in Affymetrix Cluster 2.3.**

**Fig S19. Results of DAVID functional enrichment analysis for genes in Affymetrix Cluster 5.**

## Illumina Highly Selected Clusters

Similarly to analyses for Affymetrix data, we investigated more closely the clusters which had at least one gene from every Illumina model. The results are presented in Figs S21 to S24 (for an in-depth analysis see the main manuscript).


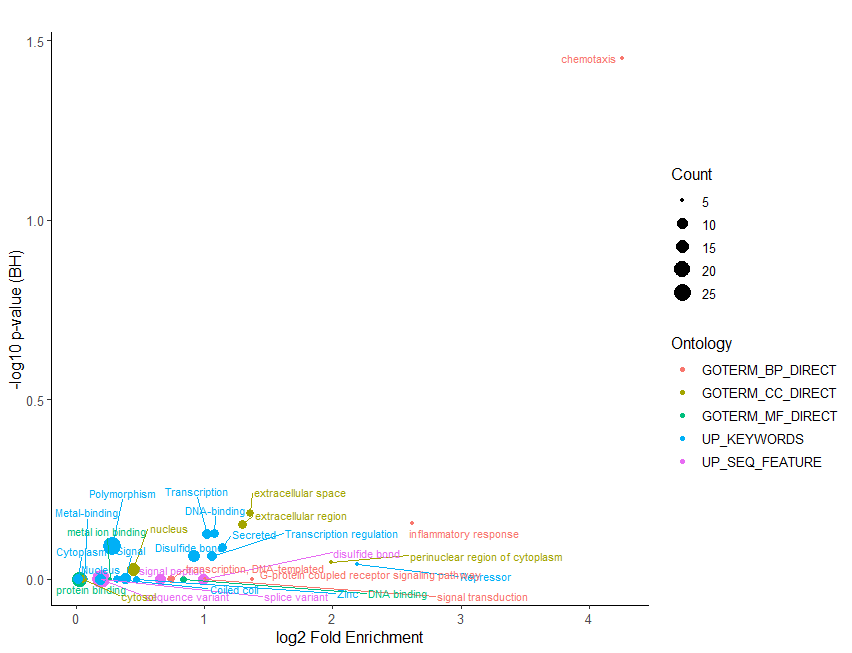


**Fig S20. Results of DAVID functional enrichment analysis for genes in Illumina Cluster 3.1.3.**


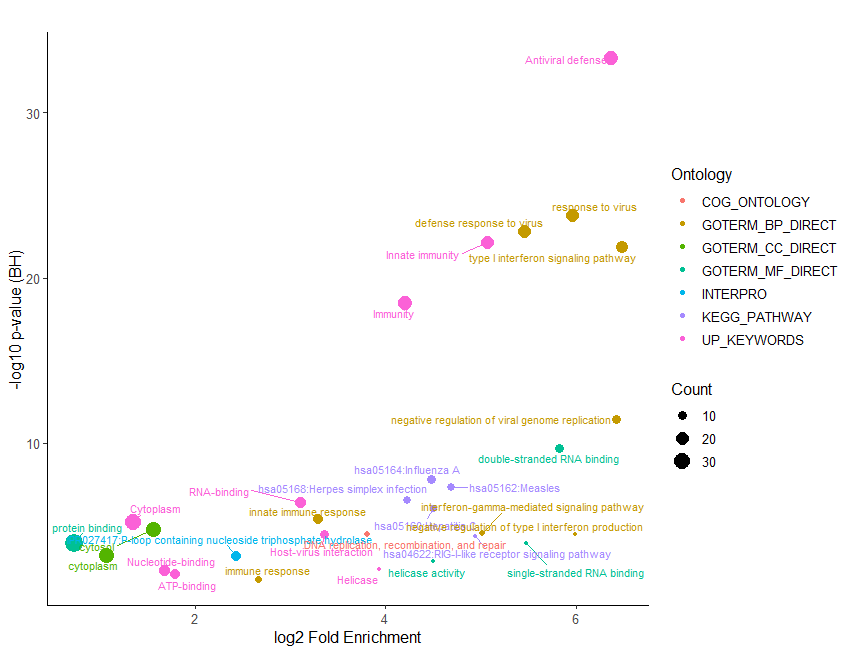


**Fig S21. Results of DAVID functional enrichment analysis for genes in Illumina Cluster 3.1.4.**


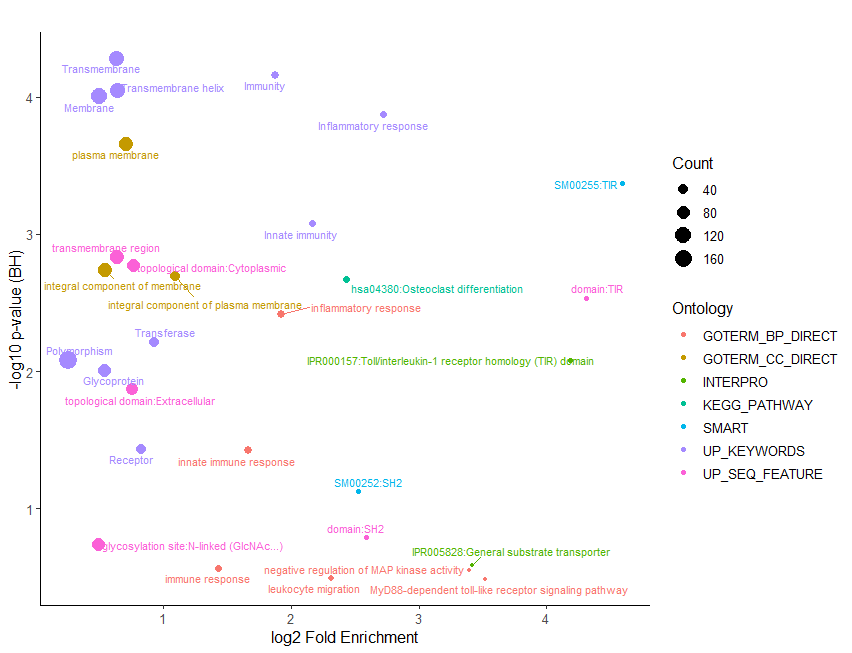


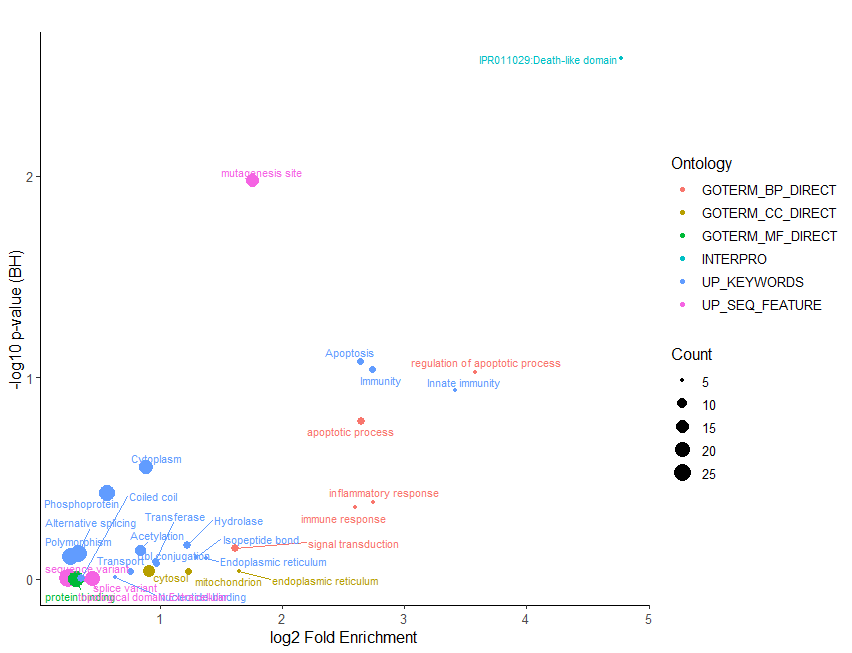


**Fig S22. Results of DAVID functional enrichment analysis for genes in Illumina Cluster 3.1.5.**

**Fig S23. Results of DAVID functional enrichment analysis for genes in Illumina Cluster 3.4.**

# S4 Out-sample results for study gene lists

**Table S23. Optimal model performance on out of sample data**

|  | Model- Dataset Combination | Out-sample Dataset | Balanced Accuracy (B/C/V) | Sensitivity (B/C/V) | Specificity (B/C/V) | Mcnemar’s Test p-value |
| --- | --- | --- | --- | --- | --- | --- |
| Affymetrix  Identified Models | BW_C | Illumina-C | 0.88 / 0.73/ 0.82 | 0.83 / 0.54/ 0.89 | 0.94 / 0.92/ 0.76 | 4.1e-9 |
|  | BW_I | Illumina-C | 0.89 / 0.73/ 0.83 | 0.83 / 0.54/ 0.89 | 0.94 / 0.93/ 0.76 | 2.3e-17 |
|  | GA_C | Illumina-C | 0.85 / 0.71/ 0.80 | 0.78 / 0.50/ 0.88 | 0.93 / 0.92/ 0.72 | 2.1e-14 |
|  | GA_I | Illumina-C | 0.88 / 0.74/ 0.83 | 0.82 / 0.55/ 0.88 | 0.94 / 0.92/ 0.77 | 0.0025 |
| Illumina  Identified Models | BW_C | Affymetrix-C | 0.77 / 0.75/ 0.80 | 0.56 / 0.57/ 0.95 | 0.98 / 0.94/ 0.65 | 0.0027 |
|  | BW_I | Affymetrix-C | 0.71 / 0.77/ 0.80 | 0.44 / 0.59/ 0.96 | 0.98 / 0.95/ 0.65 | 6.1e-12 |
|  | GA_C | Affymetrix-C | 0.72 /0.77/ 0.81 | 0.47 / 0.61/ 0.94 | 0.98 / 0.94/ 0.67 | 0.0098 |
|  | GA_I | Affymetrix-C | 0.73 /0.79/ 0.81 | 0.47 / 0.63/ 0.96 | 0.98 / 0.95/ 0.67 | 0.0024 |
|  | Average | - | 0.80 /0.75/ 0.81 | 0.65 /0.57/ 0.92 | 0.96 / 0.93/ 0.71 | 0.0021 |

**Table S24 Confusion matrix of Affymetrix identified model BW_C on Illumina-C data**

|  | | Reference | | |
| --- | --- | --- | --- | --- |
|  |  | Bacterial | Control | Viral |
| Prediction | Bacterial | 119 | 26 | 10 |
|  | Control | 7 | 105 | 38 |
|  | Viral | 18 | 64 | 373 |

**Table S25 Confusion matrix of Affymetrix identified BW_I model on Illumina-C data.**

|  | | Reference | | |
| --- | --- | --- | --- | --- |
|  |  | Bacterial | Control | Viral |
| Prediction | Bacterial | 120 | 27 | 10 |
|  | Control | 5 | 105 | 35 |
|  | Viral | 19 | 63 | 376 |

**Table S26 Confusion matrix of Affymetrix identified GA_C model on Illumina-C data.**

|  | | Reference | | |
| --- | --- | --- | --- | --- |
|  |  | Bacterial | Control | Viral |
| Prediction | Bacterial | 112 | 27 | 15 |
|  | Control | 9 | 97 | 37 |
|  | Viral | 23 | 71 | 369 |

**Table S27 Confusion matrix of Affymetrix identified GA_I model on Illumina-C data.**

|  | | Reference | | |
| --- | --- | --- | --- | --- |
|  |  | Bacterial | Control | Viral |
| Prediction | Bacterial | 118 | 27 | 11 |
|  | Control | 8 | 108 | 371 |
|  | Viral | 18 | 60 | 371 |

**Table S28 Confusion matrix of Illumina identified BW_C model on Affymetrix-C data**

|  | | Reference | | |
| --- | --- | --- | --- | --- |
|  |  | Bacterial | Control | Viral |
| Prediction | Bacterial | 19 | 5 | 2 |
|  | Control | 7 | 56 | 11 |
|  | Viral | 8 | 38 | 252 |

**Table S29 Confusion matrix of Illumina identified BW_I model on Affymetrix-C data.**

|  | | Reference | | |
| --- | --- | --- | --- | --- |
|  |  | Bacterial | Control | Viral |
| Prediction | Bacterial | 15 | 6 | 3 |
|  | Control | 7 | 58 | 8 |
|  | Viral | 12 | 35 | 254 |

**Table S30 Confusion matrix of Illumina identified GA_C model on Affymetrix-C data.**

|  | | Reference | | |
| --- | --- | --- | --- | --- |
|  |  | Bacterial | Control | Viral |
| Prediction | Bacterial | 16 | 6 | 3 |
|  | Control | 7 | 60 | 12 |
|  | Viral | 11 | 33 | 250 |

**Table S31 Confusion matrix of Illumina identified GA_I model on Affymetrix-C data.**

|  | | Reference | | |
| --- | --- | --- | --- | --- |
|  |  | Bacterial | Control | Viral |
| Prediction | Bacterial | 16 | 5 | 2 |
|  | Control | 6 | 62 | 9 |
|  | Viral | 12 | 32 | 254 |

# S5 Testing published gene list on batch corrected data

We evaluated the performance of a previously published gene list by *Sweeney et al.* [12] as was done with our out-sample optimal model, by training and testing a model with the same feature set on 60/40 data split for both Affymetrix and Illumina data. This published gene list contained seven genes (*IFI27, JUP, LAX1, HK3, TNIP1, GPAA1, and CTSB*) reported as being able to discriminate between of bacterial and viral infections. Of these seven genes both *IFI27* and *HK3* were also highlighted as significant by our analysis, indicated by inclusion amongst top models, and in the gene selection frequency analysis (S2 Appendix and Table S39).

We found the seven gene biomarker list performance varied both by class-based metrics, and also between manufacturer datasets (Table S32). Contrary to findings in [12] we found that the Sweeney gene list-derived model had a relatively higher sensitivity for virial infections (viral sensitivity: 0.94 in Affymetrix C, and 0.85 in Illumina C; Table S32) and lower for bacterial infections (bacterial sensitivity: 0.11 in Affymetrix C, and 0.61 in Illumina C)(Table S32) when tested on the data sets in our set. In terms of balanced accuracy, bacterial classes performed worse in the Affymetrix C set (0.55), whereas on the Illumina data, bacterial balanced accuracy was greater (0.78), in fact this was also greater than both control and viral balanced accuracy (0.73, and 0.75 respectively) (Table S32). To further compare the models we recalculated the score derived by *Sweeney et al* on the Affymetrix and Illumina datasets (Fig S25). More specifically the score in the paper is described as:

$$Score=\left( \left( \prod_{i=1}^{viral} x_{i} \right)^{\frac{1}{viral}}-\left( \prod_{j=1}^{bacterial} x_{j} \right)^{\frac{1}{bacterial}} \right)*\frac{viral}{bacterial}$$

Where bacterial and viral represent the number of utilised genes within the score. For Sweeney et al this equated to 3 viral associated genes (IFI27, JUP, and LAX1) and 4 bacterial associated genes (HK3, TNIP1, GPAA1, and CTSB). By applying this score to our dataset we found that in Affymetrix on average stratification was possible while in the Illumina dataset it showed limited ability.

**Table S32 Performance metrics for Sweeney et al published gene list on Affymetrix-c and Illumina-c Dataset.**

|  | Balanced Accuracy (B/C/V) | Sensitivity  (B/C/V) | Specificity (B/C/V) | Mcnemar’s Test p-value |
| --- | --- | --- | --- | --- |
| Affymetrix-c | 0.55 / 0.71 / 0.69 | 0.11 / 0.49 / 0.94 | 0.99 / 0.93 / 0.44 | 1.166e-08 |
| Illumina-c | 0.78 / 0.73 / 0.75 | 0.61 / 0.57 / 0.85 | 0.95 / 0.88 / 0.67 | 0.07317 |
| Average | 0.67 / 0.72 / 0.72 | 0.36 / 0.53 / 0.90 | 0.97 / 0.91 / 0.56 | 0.0366 |

**Table S33 Confusion matrix for Sweeney et al published gene list on Affymetrix Dataset.**

|  | | Reference | | |
| --- | --- | --- | --- | --- |
|  |  | Bacterial | Control | Viral |
| Prediction | Bacterial | 4 | 2 | 1 |
|  | Control | 4 | 49 | 16 |
|  | Viral | 26 | 48 | 248 |

**Table S34 Confusion matrix for Sweeney et al published gene list on Illumina Dataset.**

|  | | Reference | | |
| --- | --- | --- | --- | --- |
|  |  | Bacterial | Control | Viral |
| Prediction | Bacterial | 46 | 16 | 14 |
|  | Control | 7 | 110 | 52 |
|  | Viral | 22 | 66 | 370 |

**Table S35 Performance metrics for Sweeney et al published gene list on Affymetrix-I and Illumina-I Dataset**

|  | Balanced Accuracy (B/C/V) | Sensitivity  (B/C/V) | Specificity (B/C/V) | Mcnemar’s Test p-value |
| --- | --- | --- | --- | --- |
| Affymetrix-i | 0.85 / 0.65 / 0.77 | 0.72 / 0.31 / 0.97 | 0.97 / 0.99 / 0.56 | 2.463e-07 |
| Illumina-i | 0.83 / 0.72 / 0.79 | 0.71 / 0.52 / 0.89 | 0.94 / 0.92 / 0.69 | 0.00051 |
| Average | 0.84 / 0.69 / 0.78 | 0.72 / 0.42 / 0.93 | 0.96 / 0.96 / 0.63 | 0.00026 |

**Table S36 Confusion matrix for Sweeney et al published gene list on Affymetrix-I Dataset.**

|  | | Reference | | |
| --- | --- | --- | --- | --- |
|  |  | Bacterial | Control | Viral |
| Prediction | Bacterial | 44 | 2 | 5 |
|  | Control | 1 | 16 | 2 |
|  | Viral | 16 | 33 | 216 |

**Table S37 Confusion matrix for Sweeney et al published gene list on Illumina-I Dataset.**

|  | | Reference | | |
| --- | --- | --- | --- | --- |
|  |  | Bacterial | Control | Viral |
| Prediction | Bacterial | 52 | 12 | 5 |
|  | Control | 4 | 54 | 18 |
|  | Viral | 17 | 38 | 178 |

**Fig S24 - Geometric mean of expressions by sample class and batch corrected dataset (Affymetrix-C and Illumina-C).** The score derived by Sweeney et al. appears to separate bacterial and viral samples on average but fails to distinguish control samples. Large 25% and 75% quantiles also suggest this simple geneset misclassifies samples easily. Depending on the technology used a different threshold seems to be required for optimal classification.

**Fig S25 - Geometric mean of expressions by sample class and batch corrected dataset (Affymetrix-I and Illumina-I).** The score derived by Sweeney et al. appears to separate bacterial and viral samples on average but fails to distinguish control samples. Large 25% and 75% quantiles also suggest this simple geneset misclassifies samples easily. Similarly to the confirmed dataset above, technology dependent thresholds for the score need to be set to optimally predict the samples.

# S6 External Files

**Figure S26.pdf** - **Affymetrix Interaction Network.** Affymetrix recovered interaction network at first level of clustering. Selected model genes are highlighted.

**Figure S27.pdf** - **Illumina Interaction Network.** Illumina recovered interaction network at first level of clustering. Selected model genes are highlighted.

**Table S38** – **Model Gene Selection Frequency.** Affymetrix and Illumina model selected genes with relative frequency of selection (genes with greater than 5% aggregated inclusion across all search procedures).

**Table S39** – **Highly selected gene clusters from Affymetrix and Illumina interaction network.** Table containing the genes from the 4 highly model selected Illumina clusters, and 5 highly model selected gens from the Affymetrix clusters.

# Bibliography

1. Johnson, W.E., C. Li, and A. Rabinovic, *Adjusting batch effects in microarray expression data using empirical Bayes methods.* Biostatistics, 2007. **8**(1): p. 118-27.

2. Fisher, R.A., *Statistical methods for research workers*, in *Breakthroughs in statistics*. 1992, Springer. p. 66-70.

3. Falcon, S. and R. Gentleman, *Hypergeometric Testing Used for Gene Set Enrichment Analysis*, in *Bioconductor Case Studies*. 2008, Springer New York: New York, NY. p. 207-220.

4. Pearson, K., *LIII. On lines and planes of closest fit to systems of points in space.* The London, Edinburgh, and Dublin Philosophical Magazine and Journal of Science, 1901. **2**(11): p. 559-572.

5. Huang, D.W., et al., *The DAVID Gene Functional Classification Tool: a novel biological module-centric algorithm to functionally analyze large gene lists.* Genome biology, 2007. **8**(9): p. R183-R183.

6. Blaha, J., et al., *High-level expression and purification of soluble form of human natural killer cell receptor NKR-P1 in HEK293S GnTI(-) cells.* Protein Expr Purif, 2017. **140**: p. 36-43.

7. Chen, C., et al., *LNMAT1 promotes lymphatic metastasis of bladder cancer via CCL2 dependent macrophage recruitment.* Nature Communications, 2018. **9**(1): p. 3826.

8. Stanton, G.J., et al., *Interferon review.* Invest Radiol, 1987. **22**(3): p. 259-73.

9. Wigington, C.P., et al., *Poly(A) RNA-binding proteins and polyadenosine RNA: new members and novel functions.* Wiley Interdiscip Rev RNA, 2014. **5**(5): p. 601-22.

10. Boxx, G.M. and G. Cheng, *The Roles of Type I Interferon in Bacterial Infection.* Cell host & microbe, 2016. **19**(6): p. 760-769.

11. Schroeder, H.W., Jr. and L. Cavacini, *Structure and function of immunoglobulins.* The Journal of allergy and clinical immunology, 2010. **125**(2 Suppl 2): p. S41-S52.

12. Sweeney, T.E., H.R. Wong, and P. Khatri, *Robust classification of bacterial and viral infections via integrated host gene expression diagnostics.* Sci Transl Med, 2016. **8**(346): p. 346ra91.
