# Supplementary figures and images for: An OMICs-based meta-analysis to support infection state stratification
Source: Bioinformatics. 2021 Feb 9;37(16):2347–55. doi: 10.1093/bioinformatics/btab089 (PMC8388022; doi:10.1093/bioinformatics/btab089)

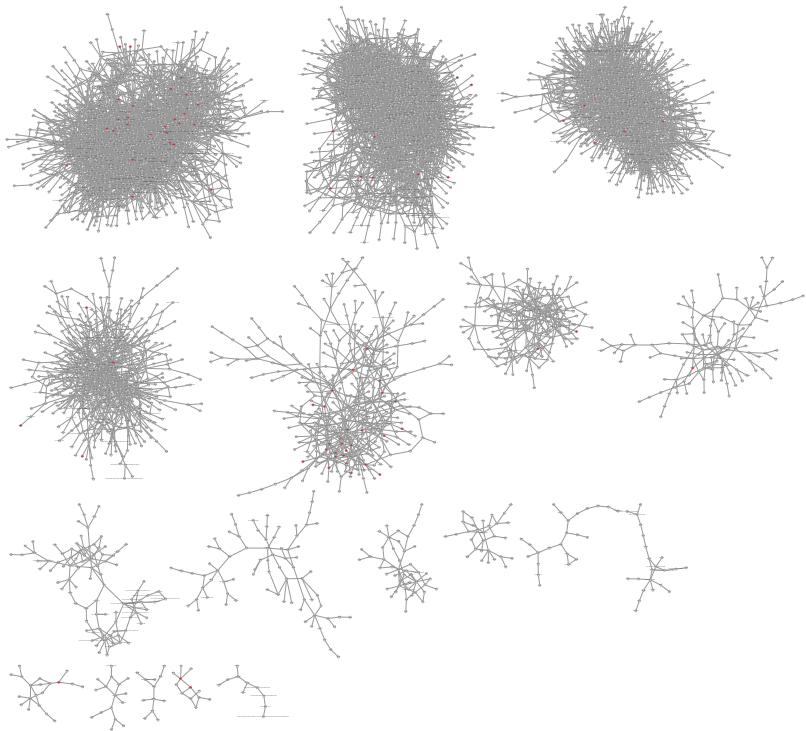

Supplement: btab089_Supplementary_Data [file btab089_supplementary_data.zip › Figure S26.pdf]

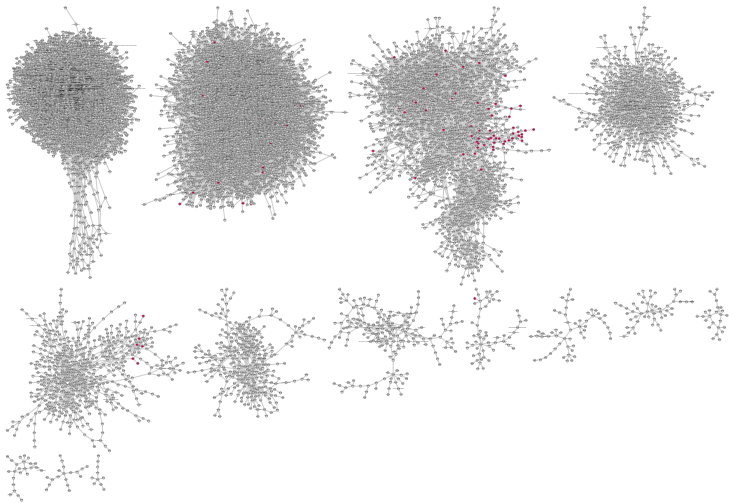

Supplement: btab089_Supplementary_Data [file btab089_supplementary_data.zip › Figure S27.pdf]
